# Microcomb-enabled parallel self- calibration optical convolution streaming processor

**DOI:** 10.1038/s41377-025-02093-5

**Published:** 2026-03-05

**Authors:** Jiajia Wang, Xingyuan Xu, Xiaotian Zhu, Yifu Xu, Shifan Chen, Haoran Zhang, Yixuan Zheng, Shuying Li, Yunping Bai, Zhihui Liu, Roberto Morandotti, Brent E. Little, Sai T. Chu, Arthur J. Lowery, David J. Moss, Kun Xu

**Affiliations:** 1 State Key Laboratory of Information Photonics and Optical Communications, Beijing University of Posts and Telecommunications, Beijing, China; 2 Department of Physics, City University of Hong Kong, Tat Chee Avenue, Hong Kong, China; 3 INRS-Énergie, Matériaux et Télécommunications, 1650 Boulevard Lionel-Boulet, Varennes, QC J3X 1S2 Canada; 4 QXP Technology Inc., Xi’an, 710119 China; 5 Electro-Photonics Laboratory, Department of Electrical and Computer Systems Engineering, Monash University, Clayton, VIC 3800 Australia; 6 Optical Sciences Centre, Swinburne University of Technology, Hawthorn, VIC 3122 Australia

**Keywords:** Integrated optics, Optoelectronic devices and components

## Abstract

The exponential growth of cloud computing and artificial intelligence (AI) applications has driven an urgent need for high-bandwidth, energy-efficient hardware architectures in data centers. With Moore’s Law nearing its limits, optical neuromorphic computing hardware offers a promising alternative, providing ultra-high speeds and minimal energy consumption due to its analog architecture. Here, we propose the microcomb-enabled parallel optical convolution streaming processor (OCSP) with time, space, and wavelength three-dimensional multiplexing, operating at data rates of 50 GBaud or higher, achieving a convolution computing speed of up to 4 trillion operations per second (TOPS). Moreover, the OCSP uses a robust self-calibration mechanism to achieve accurate optical phase calibration and set-up of its convolution function. This innovative approach leverages time-space interleaving passive periodic interference architecture, incorporating wavelength-division-multiplexing technology, and is verified experimentally for parallel image feature extraction and recognition tasks. Our OCSP offers a practical pathway for seamlessly integrating photonic computing units into data center interconnects, unlocking photonic computing’s potential for scalable, low-latency AI workloads.

## Abstract (web summary)

Parallel optical convolution streaming processor: we demonstrate the parallel optical convolution streaming processor, incorporating microcomb-enabled wavelength-division-multiplexing technology, achieving a parallel convolution computing speed of up to 4 trillion operations per second.

## Graphical abstract

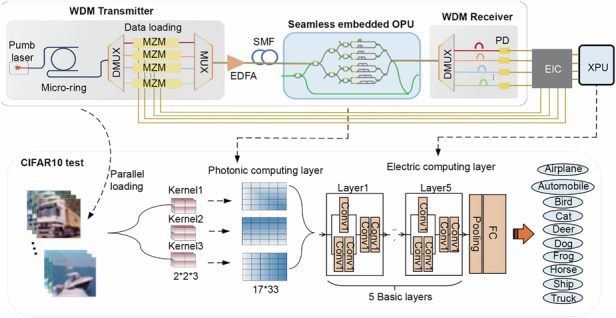

## Introduction

In recent years, with the rapid development of information technologies such as cloud computing, artificial intelligence (AI), and big data, the data volume and computing power demands of data centers have witnessed explosive growth. The rapid development of computationally intensive technologies has generated a critical need for energy-efficient and cost-effective data center interconnects and intelligent computing hardware that can accommodate substantial capacity^1–7^. Firstly, optical interconnection, characterized by its high bandwidth capabilities, facilitates rapid data transmission and addresses the pressing demand for increased bandwidth in data centers. Furthermore, Wavelength Division Multiplexing (WDM) technology enables the simultaneous transmission of multiple optical signals at varying wavelengths over a single fiber, thereby significantly enhancing the available bandwidth for data transfer and accommodating the exponential growth of data volume. Chip-scale microcombs have emerged as a transformative solution for WDM systems, owing to their unique combination of octave-spanning spectral coverage (>100 nm) and stable high-repetition-rate modes (typically 10–100 GHz)^8,9^. Recent advancements have showcased the capability of massively parallel data transmission utilizing various types of microcomb sources^10–13^.

Secondly, optical neuromorphic computing hardware^14–40^, offering ultra-high speeds facilitated by the >10 THz wide optical bandwidth together with, minimal energy consumption down to 2.5 × 10^−19^ J per operation due to their analog architecture^14,15^, are promising candidates for hardware accelerators. While digital electronics generally perform convolutions via Fourier transforms (as convolution in the time domain is equivalent to multiplication in the frequency domain) to reduce the computing complexity from O(n^2^) to O(nlog(n)), analog optics can accelerate convolutions in a dramatically different way—simply following the time domain definition that involves splitting the signal into several paths, applying weights and delays, then recombining. These analog methods are suited to convolution acceleration, since on the one hand, the size of typical convolutional kernels (i.e., 3 × 3) is well within the capability/parallelism of analog optical systems; on the other hand, analog approaches promise: a) much lower energy consumption, as computation is performed during transmission, thus eliminating the data read/write processes in von Neumann hardware; b) streaming data processing at higher bandwidths throughputs reaching over 100 GBaud that enables real-time low-latency processing of large-volume optical data streams^41^, and parallelism using WDM. While significant progress has been made in optical convolution hardware that exploits wavelength-division-multiplexing parallelisms^25,28^, phase-changing materials^17^, and higher computing dimensions^29^, there still remain substantial opportunities for significant improvements. Existing approaches encounter substantial challenges in the further expansion of computing parallelism, ultra-high-speed data loading, and stable weight configuration. For example, the microcomb-based non-coherent computing architecture, although microcomb provides broad bandwidth (>100 nm) with dozens of wavelength channels, the computational scalability is ultimately constrained by reconfigurable component quantity for each additional comb line requiring a corresponding micro-ring resonator (MRR) unit. However, the integration scale of the MRR is physically constrained by its limited free spectral range (FSR). Furthermore, as the scale of integration increases, issues such as drift in MRR resonances and inter-channel crosstalk will become increasingly pronounced. This renders the systems highly susceptible to environmental perturbations, thereby complicating the stability control of the micro-ring weights even further^28–31^. The coherent optical computing architecture based on the Mach-Zehnder interferometer (MZI) mesh with multi-input multi-output (MIMO) port, high-speed information loading is accompanied by signal synchronization issues. Additionally, the number of basic MZI units in coherent computing architectures usually grows quadratically with the increase in the number of input ports, which poses significant challenges for chip reconfiguration^16,39^.

Passive and time-space interleaving optical convolution operation units are key enablers for the ultra-high-speed data loading and high-parallelism optical convolution computing, as they specifically enable: a) the coherent optical convolution computing architecture characterized by single-input and single-output, the challenges associated with high-speed signal synchronization in MIMO systems are effectively avoided. b) decoupling wavelength from on-chip components establishes two independent scaling dimensions: the OCSP chip’s physical pathways scale convolutional kernel size without wavelength constraints; wavelength channels independently extend input data dimensionality.

However, challenges in accurate phase control and alignment hinder coherent computing operations in existing optical convolution hardware (multi-wavelength sources^17,25,28^ and external electronics^29^ are used instead), due to the difficulties in calibrating and manipulating the optical phase sufficiently accurately, that is, on optical frequency scale rather than a microwave-signal scale^42^. This precludes using on-chip signal interference and thus efficient use of spatial-division parallelism — the powerful and scalable computing parallelism.

Here, we propose and demonstrate a microcomb-enabled high-parallel optical convolutional streaming processor (OCSP) with key features including: a) wavelength-division multiplexing, time-space interleaving architecture operating at a symbol rate of 50 GBaud with the potential to go much higher. b) The computing speed achieves 4 trillion operations per second (TOPS). (i.e., with reconfigurable synaptic weights), representing the highest computing speed for integrated optical convolution hardware accelerators c) built-in reference waveguide for on-chip optical phase and amplitude weight calibration enabling accurate setting of the synaptic weights. The OCSP is achieved by coherently interleaving the time and space; simultaneously, it incorporates wavelength-division multiplexing technology to support the large-scale data requirements for both speed and capacity. Its performance is experimentally verified by implementing generic image feature extraction functions and image recognition tasks. The OCSP demonstrates significant potential as a universal convolutional front-end embeddable in data centers, effectively enhancing computational throughput while reducing energy consumption.

## Results

### Principle of operation

**Fig. 1:**
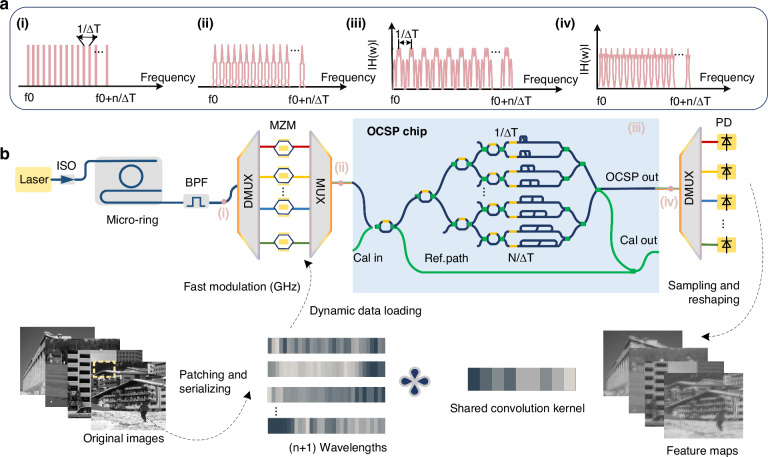
Parallel optical convolution streaming processing system. **a** Optical spectra at critical locations of the parallel optical convolution streaming processing system. (i) the microcomb with a repetition rate of 1/ΔT serves as a multi-wavelength source. (ii) output optical spectrum of the modulated multi-wavelength signal, where each carrier is independently loaded with data at a symbol rate of 1/ΔT. (iii) the OCSP’s transmission spectrum with a free spectral range (FSR) of 1/Δ*T*. (iv) multi-wavelength data streams after parallel convolution processing by the OCSP. **b** System diagram for parallel image processing using the OCSP. Multiple image data streams are independently loaded onto distinct wavelength channels and transmitted through a shared optical fiber to the OCSP chip, enabling parallel convolution processing. Wavelength-selective switches can be employed to dynamically manage access to these data streams. The outputs are then demultiplexed for simultaneous, parallel readout of all processed streams. ISO optical isolator, BPF bandpass filter, DMUX demultiplexer, MUX multiplexer, MZM Mach-Zehnder modulator, PD photodetector

The core structure integrated in the photonic chip comprises: a passive streaming processing unit, a reference waveguide, and two pairs of fan-out for data processing and calibration respectively (Fig. 1b). The streaming processing unit can be configured as a finite impulse response (FIR) filter which can be modeled as a finite impulse filter^42^. Mathematically, the transfer function in the time and frequency domain can be given by:

$$H\left(f\right)={\sum }_{n=0}^{N-1}{a}_{n}{e}^{j{\varphi }_{n}}{e}^{-j2\pi {fn}\Delta T}$$

$$h\left(t\right)={\sum }_{n=0}^{N-1}{a}_{n}{e}^{j{\varphi }_{n}}\delta (t-n\Delta T)$$

Where *f* is the optical frequency, *N* denotes the number of taps and *n*Δ*T* is the delay of tap *n*, the amplitude and phase of the tap are represented by ${a}_{n}$ and ${\varphi }_{n}$ and can be fully adjusted in all dimensions by changing the splitting ratio of the MZI and the phase shift of the phase shifter. The *H*(*f*) is periodic with a FSR of 1/Δ*T* (FSR = 1/Δ*T*). Benefiting from the periodic interference characteristics of the filter, when the carrier frequency spacing of the input streams aligns with the filter’s FSR, the OCSP can simultaneously perform parallel convolution across multiple data streams, without necessitating additional on-chip components (as shown in Fig. 1a, b).

For an input optical data streams, the filter output is given by the convolution:

$$y\left(t\right)=x\left(t\right)* h\left(t\right)={\sum }_{n=0}^{N-1}{a}_{n}{e}^{j{\varphi }_{n}}x(t-n\Delta T)$$

The input optical data streams, at a symbol rate 1/ΔT Baud (1/ΔT is also the repetition frequency of microcomb), are wavelength-division multiplexed and feature unlimited lengths for streaming processing. The input optical data streams are simultaneously duplicated and weighted via cascaded MZIs, with the phases controlled by phase shifters (PSs). Next, the weighted replicas are progressively delayed with a delay step (between adjacent spatial paths) equals to ΔT, the symbol duration of input data streams, effectively achieving time-space interleaving (i.e., the input data’s adjacent symbols are aligned in time). Finally, the weighted and delayed replicas are coupled together, constructively or destructively interfering with each other to yield convolution results.

The convolution window effectively slides at the input data rate. Each output symbol is the dot product between the input data, within a convolution window or receptive field (determined by the scale of the chip, i.e., number of spatial paths *N*), and the weights (implemented by the MZIs and PSs). Consequently, each output symbol is the result of *N* multiply-and-accumulate (MAC) operations. The OCSP directly processes the optical fields when the OCSP processes real-valued data (i.e., intensity-modulated, phases are 0 or π), each of which takes 2 operations (1 multiplication and 1 accumulation) and thus results in a peak computing speed of 2 *N*/Δ*T* operations per second (OPS), per wavelength channel. This computing speed can be boosted when processing massive wavelength-division multiplexed optical data streams; and the frequency interval between wavelength channels needs to be equal to, or multiple of, the symbol rate 1/Δ*T*.

### Kernel self-calibration of the OCSP

**Fig. 2:**
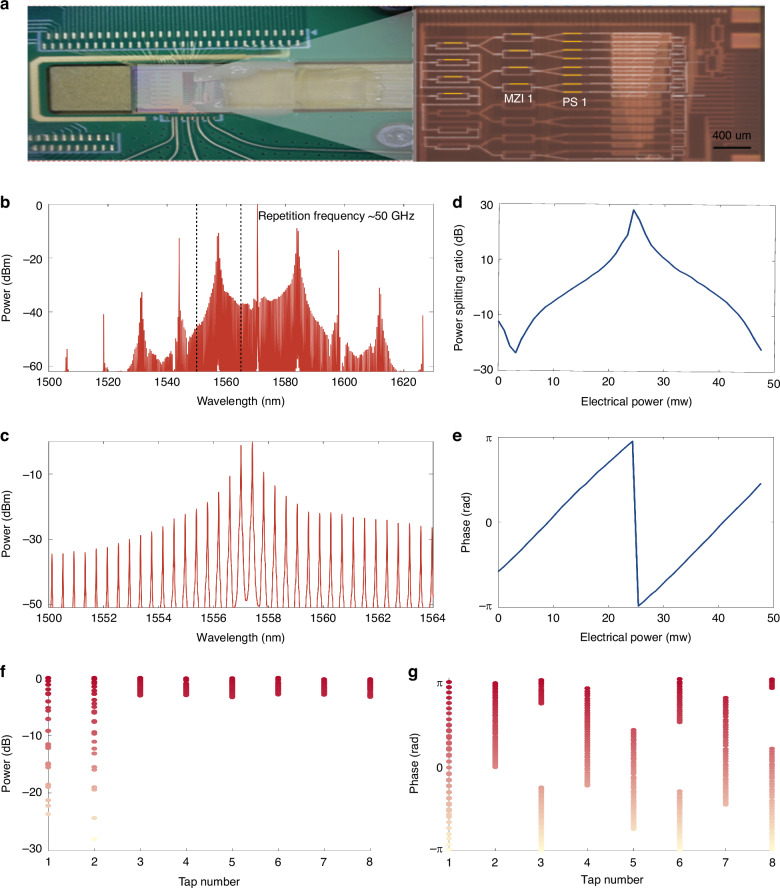
Chip fabrication and characterization. **a** Photograph of the packaged OCSP chip. Optical signals enter and leave the chip via an edge-coupled fiber array. **b** The spectrum of the microcomb, with a repetition frequency of ~50 GHz. **c** The zoom-in of microcomb. **d** The extracted relationship between the applied electrical power and the power splitting ratio of the MZI. **e** The extracted relationship between the applied electrical power and the phase shifts of the phase shifters. **f** The recovered power of the taps with sweeping electrical power applied onto MZI1. **g** The recovered phases of the taps with sweeping electrical power applied onto phase shifter1

**Fig. 3:**
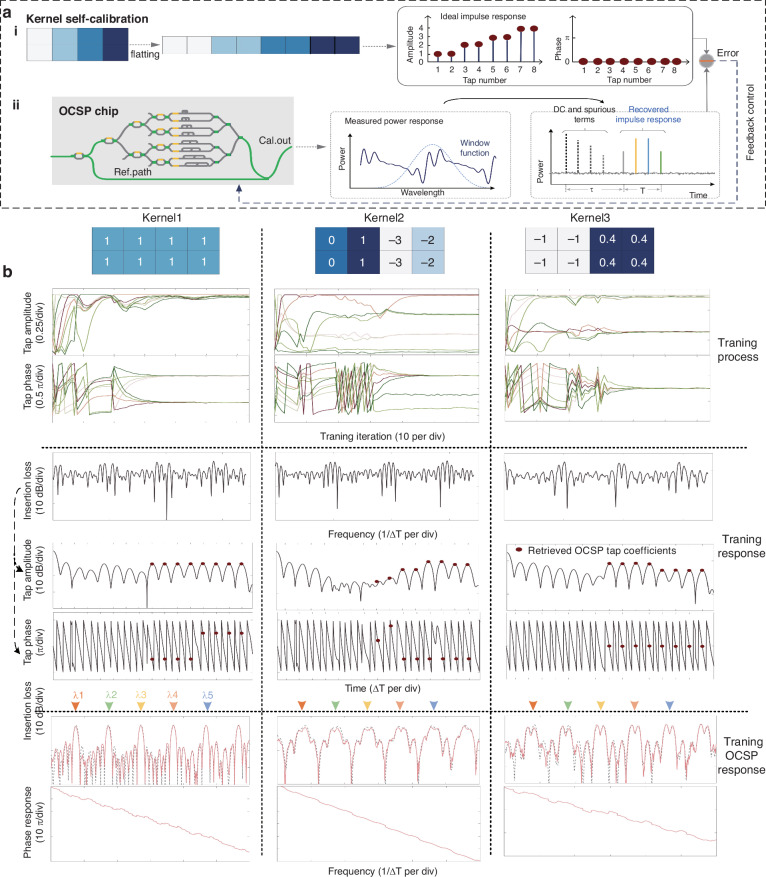
Calibrating processes of OCSP implementing three kernel settings. **a** Kernel self-calibrating schematic diagram. **b** The convergence curve of the OCSP taps’ amplitudes (first row) and phases (second row) within 65 iterations. The power response (third row) and impulse responses (obtained via Fourier transform, fourth and fifth rows) of the trained OCSP chip with the reference path included (i.e., the power response measured via the calibration ports). The frequency responses of the trained OCSP without the reference path (i.e., the power response measured via the signal ports, last two rows). λ_1_ = 1556.66 nm, λ_2_ = 1557.07 nm, λ_3_ = 1557.48 nm, λ_4_ = 1557.89 nm and λ_5_ = 1558.3 nm denotes the carrier of input wavelength-division multiplexed data streams

The key of the wavelength-division multiplexing and time-space interleaving convolutional computing architecture is rooted in two factors: a) the alignment of the optical comb repetition frequency with the FSR of the OCSP, and b) the alignment of phase shifts induced by each path on the OCSP. Yet any tiny delay variations in the magnitude of optical wavelengths (~1550 nm) can lead to large phase errors in the convolution weights and thus in the computing results. This is a nontrivial issue due to the challenges in obtaining phase-related responses of the chip and overcoming the dynamic on-chip thermal crosstalk or temperature disturbance (Fig. 2f, g). To address this, we first map the convolution kernel to the time-domain taps of the OCSP chip. Subsequently, we apply an on-chip phase recovery method^43^ to optical computing chips (Fig. 3a). This adds an optical reference path to the chip, which enables phase recovery based on the intensity-only spectral response of the chip via the gap method. The intensity response can be easily measured with an external tunable laser source and a power meter; by placing the reference path on the chip we ensure that any phase errors in the patch that lead to external instrumentation do not affect the phase measurement. The measured power response of the entire OCSP chip can be regarded as a superposition of spectral components yielded by “internal” (between the streaming processing taps) and “external” (between the reference tap and the streaming processing taps) interferences. As such, the impulse response of the OCSP (i.e., the convolutional weights) can be obtained via the Fourier transform of the entire chip’s power response, provided the reference path is sufficiently shorter than the streaming processing paths (τ > *T*). With the dynamic convolutional weights obtained (both the amplitudes and phases), the required updates of the electrical power supply can be obtained and the desired kernel weights can be deterministically dialed-up (for the detailed self-calibration process and robustness test, see Supplementary Notes 4, 5). Beyond successfully calibrating our OCSP, this phase recovery approach enables optical computing chips to obtain comprehensive dynamic on-chip frequency and impulse responses (both the phases and amplitudes), thus enabling accurate and trainable on-chip frequency responses that greatly enhances the computing accuracy and allows rapid training of coherent optical computing hardware.

### Convolutional kernel verification

To demonstrate our approach, we fabricated a 16-tap FIR chip on a standard Silicon-On-Insulator (SOI) platform^43^. Taps 9–16 were worked as the OCSP and taps 2–8 were in the off state, while tap 1 served as the reference path. The delay step of the OCSP’s streaming processing taps is Δ*T* = ~ 20 ps, corresponding to an input optical data rate of 50 GBaud. This also gives the OCSP’s impulse response duration *T* = 7 × Δ*T* = 140 ps. The delay gap between the reference path and the OCSP’s first tap is ~8 × Δ*T*, satisfying τ > *T* for subsequent phase recovery via Fourier transform. Four stages of tunable couplers were used to achieve the desired amplitudes of convolutional weights and eight PSs were used to manipulate the tap’s phases (i.e., the signs of the weights).

Distinctive convolutional kernels were first implemented using the OCSP chip for generic image processing functions (kernels 1, 2) and verification of convolution accuracy at the critical point (kernel 3) (Fig. 3b). The windowed power response of the entire chip (with ref. path) within a frequency range of 3.5/Δ*T* was Fourier transformed to obtain the impulse responses, which were then used to assess errors against the desired tap coefficients and thus the needed update of applied electrical power. After 65 iterations, the OCSP taps’ amplitudes and phases converge to their desired values and thus can be used for subsequent optical data processing (Fig. 3b, first two rows). Since the OCSP features a periodic frequency response with FSR of 1/Δ*T* (Fig. 3b, last two rows), the OCSP could support simultaneous computing/feature extraction of multiple wavelength channels, provided the wavelength channels’ spacing matches with an integer multiple of the OCSP’s FSR.

**Fig. 4:**
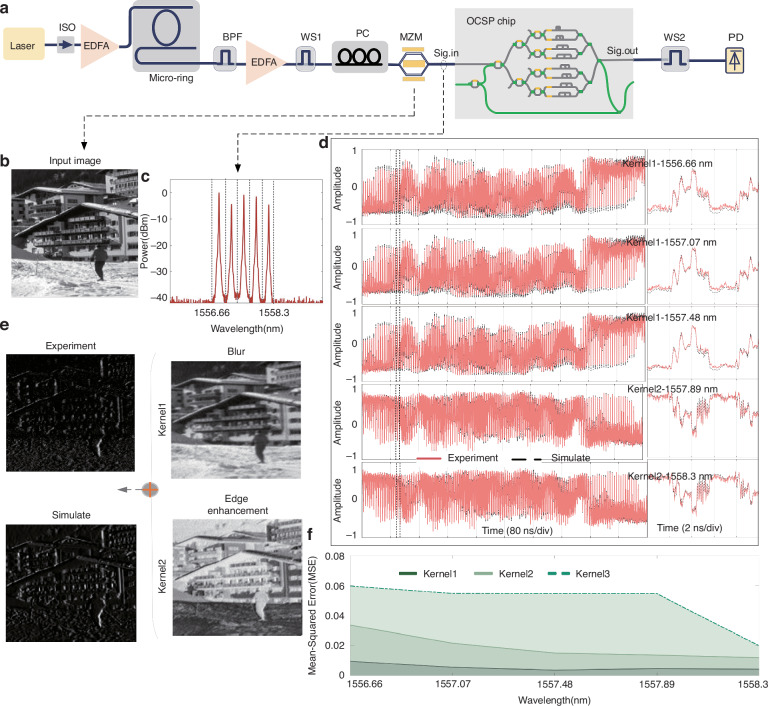
Image processing results of OCSP kernels. **a** Setup diagram. EDFA, erbium-doped fiber amplifier. BPF, bandpass filter. MZM, Mach-Zehnder modulator. PD, photodetector. **b** Original input image. **c** Spectrum diagram of five wavelengths after modulation of the signal. **d** The first column shows the experimental results of the output optical waveforms’ intensities, with the black dotted lines showing the simulated results and the second column illustrates the detailed view of the waveforms. **e** The feature maps generated after applying two different convolution kernels and the synthesized feature map (Sobel operator). **f** The convolution error curve after processing image data carried by five distinct wavelengths utilizing three different convolutional kernels

To verify the parallel computing capabilities of our OCSP chip, five wavelengths were simultaneously selected by the waveshaper1 (ws1) and transmitted through a shared optical path. The input optical data was encoded as intensities of flattened image matrices at 50 GBaud and loaded onto each wavelength channel. At the output port, the waveshaper2 (ws2) dynamically selected the computational results of each wavelength, enabling wavelength-demultiplexed readout (Fig. 4a–c). The waveforms of the convolution results and their mean squared error curves are shown in Fig. 4d, f (see Supplementary Note 2, 7 for the detailed discussion on the convolution results). The convolutional kernels directly implemented by the OCSP chip can achieve desired image processing functions including averaging (kernel 1) or edge enhancement (kernel 2). The OCSP’s convolutional weights need to satisfy certain rules such that the optical carrier can be maintained for detection: the sum of the weights corresponds to the transmission of the carrier, thus it cannot be set as zero—at which point the carrier is suppressed (i.e., located at the notches of the amplitude responses), such as the Sobel operator. We note that this does not impose any limitations onto the OCSP’s capability, since on one hand, a local oscillator laser at the same wavelength as the carrier can be offered at detection to compensate for the carrier’s power losses, on the other hand, the convolutional weights do not satisfy the rule can always be decomposed into two sets of convolutional weights that can be implemented simultaneously and then synthesized, as we have demonstrated in Fig. 4e (synthesized kernels 1 and 2). In this experiment, we break it down into two sets of taps when the sum of tap coefficients is lower than 2 (for 8-tap OCSP used) (see Supplementary Note 1, 3 for detailed discussion).

### Optoelectronic hybrid neural network

**Fig. 5:**
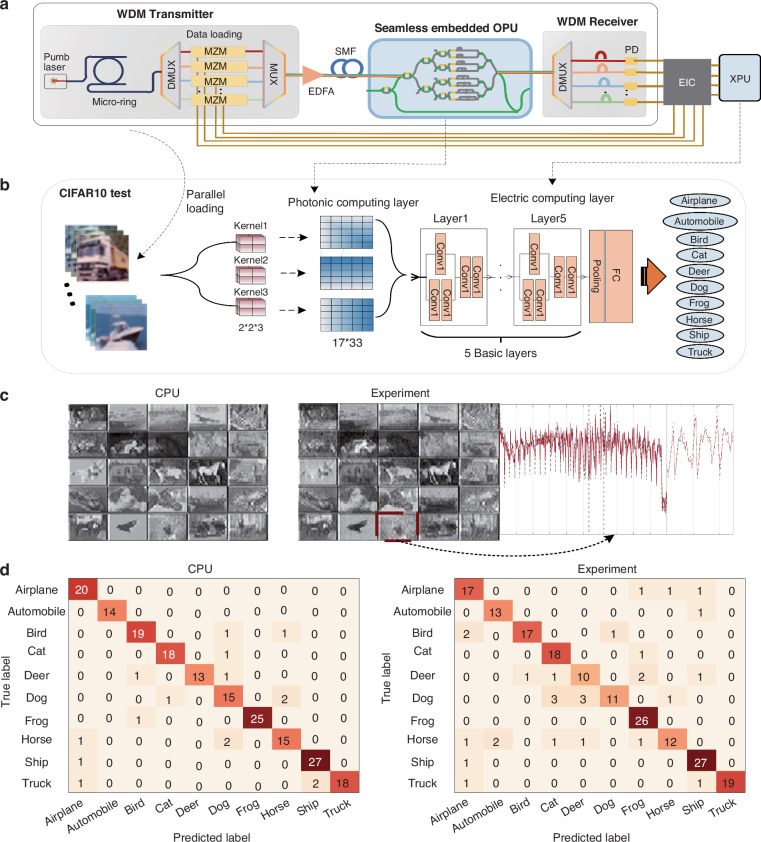
Optoelectronic hybrid neural network. **a** Schematic diagram of parallel optical interconnection in data centers. The OCSP chip is embedded in the WDM transmission link, serving as a multi-channel convolution accelerator and cooperating with electronic devices to perform complex network tasks. OPU optical processing unit. XPU accelerated Processing Unit. EIC electronic integrated circuits. **b** The architecture of the convolution neural network (CNN). Each basic layer contains four consecutive convolutional layers that form a dense processing block. To enhance gradient flow and mitigate vanishing gradients, residual connections are integrated by routing the input through a convolutional layer (for dimension alignment) before merging it with the output of the block via element-wise addition. Following these residual blocks, a pooling layer down-samples the extracted features, which are then flattened and projected onto a 10-dimensional space corresponding to the CIFAR-10 classes by the final FC layer. Conv, convolutional layer. FC, fully connected layer. **c** The experimental and simulated feature maps and waveform of kernel1. **d** The confusion matrices obtained from CPU implementation and the proposed optoelectronic hybrid architecture implementation

To further highlight the capability of the OCSP system demonstrated in this work, we performed a proof-of-concept demonstration to validate our OCSP’s deployment in the data centers and complex neural networks, thereby working in coordination with electronic hardware to facilitate advanced AI tasks. Figure 5a shows the schematics of the integrated parallel data interconnect system for large-capacity optical interconnection. At the WDM transmitter, each comb line carries independent data streams through WDM technology. The OCSP is directly embedded in the WDM transmission link, maintaining compatibility with transceiver interfaces, enabling parallel feature extraction across all wavelength channels. At the WDM receiver, the multi-channel convolutional stream is detected by the photodetector and transmitted to the next computing node after processing (such as sampling and retiming) (for detailed deployment in datacenter, see Supplementary Note 9). In this proof-of-concept study, we use microcombs as the multi-wavelength source, waveshaper and Mach-Zehnder modulators facilitate the dynamic loading of data across various wavelengths instead. We adopted the PAM-16 modulation format which is a potential enabler for next-generation data center optical interconnects, offering higher spectral efficiency than the generic PAM-4 modulation format in data centers while meeting requirements envisioned for AI workloads. To validate the universality of our OCSP chip, we conducted tests in distinct datasets. Figure 5b shows the network model in the CIFAR10^44^ data test, 200 images were divided into 5 different batches, and five different wavelengths carried the image data of different batches respectively. The input RGB image of dimensions 34 × 34 × 3 pixels was processed through three 2 × 2 × 3 convolutional kernels, generating three feature maps with spatial resolution 17 × 33 for subsequent network processing. In this experiment, the first convolutional layer was implemented using photonic computing hardware, while the subsequent layers of the network were realized through electronic computing hardware. The electronic hardware segment comprises five basic layers followed by a fully connected (FC) layer. Each basic layer contains four consecutive convolutional layers that form a dense processing block. The feature maps and the expanded waveforms of kernel 1 obtained from the experiment and the CPU are shown in Fig. 5c. The slight difference between the experiment and calculation is mainly caused by limited system bandwidth. The confusion matrix (Fig. 5d) illustrates the accuracy of the prediction obtained from experiment and theoretical calculation is 85% and 92%. (Additionally, the experiment results on ImageNet subset^45^, Fashion-MNIST^46^, and MNIST^47^ datasets are shown in Supplementary Note 10).

## Discussion

Our study further advances the potential of optical computing by introducing an OCSP featuring the WDM optical input interface. This architecture provides substantial transmission bandwidth and effectively supports high-speed, large-capacity data requirements. Moreover, it overcomes the limitation in previous optical computing architectures^28,29,33,39^: the difficulty of integrating on-chip optical computing units into optical interconnection systems due to incompatible optical input interfaces. Moreover, the OCSP chip can be fabricated using the industry-standard SOI platform, which is consistent with the platform used for commercial optical receiver modules (e.g., Intel’s 400G DR4), thereby, facilitating the co-design and integration with the optical receiver module while mitigating convolutional output errors caused by distance transmission.

The original input signal of the OCSP may experience distortion due to crosstalk and nonlinear effects in distance transmission, which could consequently introduce errors in the convolutional output. Extensive research efforts have been devoted to addressing signal distortion in data center optical interconnects. Representative approaches include MRR arrays for implementing multi-wavelength parallel dispersion compensation in the optical domain^10^, and the FIR filter-based devices for inter-symbol interference compensation^48^. Furthermore, leveraging the inherent stability of data center optical interconnect links, we can incorporate transmission link characteristics into the network training paradigm. This hardware-aware learning approach facilitates the development of adaptive neural network models that maintain robust inference accuracy under signal distortion scenarios.

The performance can be easily boosted with much higher space-/wavelength-division parallelisms and data rates: for instance, 100 GBaud’s data rate is readily available via commercial transceiver (TRXs); forty 100 GHz-spaced wavelengths in the optical C band can be offered as well by microcombs. For our passive OCSP structure, the size of the convolutional kernel is determined by the tap coefficients of the FIR filter, which corresponds to the number of delay paths. OCSP with over 64 spatial paths can be straightforwardly fabricated with passive low-loss wafer-scale nanofabrication platforms, and future designs could employ increased physical spacing between waveguide paths or integrated thermal isolation trenches in the chip substrate to alleviate thermal crosstalk. These optimizations could lead to a remarkable peak computing speed of 2 × 64 × 100 G × 40 = 0.512 Peta OPS, for a single integrated OCSP device.

In contemporary big data storage architectures, information is typically dispersed across multiple storage nodes. By facilitating the flexible allocation of wavelengths, the OCSP chip enables distributed access to extensive datasets, concurrently retrieving data from each storage node. The data center connects computing resources distributed across various locations through optical interconnects, thereby forming a virtual resource pool. The embedding of the current OCSP chip into the data center facilitates dynamic computational resource allocation with enhanced utilization efficiency. This framework enables fine-grained partitioning and distributed deployment of complex neural networks across heterogeneous computing nodes, where each node specializes in processing dedicated neural network components (e.g., input layers, hidden layers, output layers) (for more details about collaborative design with electronic control unit, see Supplementary Note 8). Through optical interconnection technology, these different computing nodes can transfer data at extremely high speeds.

## Materials and methods

### Chip fabrication and calibration

The photonic integrated circuit (PIC) was fabricated using a standard SOI platform, with a total chip footprint of 25 mm^2^. TiN heaters (each with ~1.7 kΩ resistance) are used to tune the optical phases via thermo-optical effects. The fabricated PIC is a 16-tap FIR chip. For a proof-of-concept demonstration using a standard chip design, we used the shortest path as the reference path and only half of the taps as the OCSP. The unused paths were suppressed in power and should be removed in future implementations. The dominant computational delay originates from the longest optical path (~20 mm) within the OCSP chip, yielding a total latency of 290 picoseconds. During the experiments, the chip was mounted on an external thermal controller, with the temperature stabilized at 30 °C to dissipate the heat generated by the on-chip PSs. The optical insertion loss spectra (i.e., the power response or the squared amplitude response) of the chip were measured by an optical vector network analyzer (Luna OVA 5100), which can be replaced with a wavelength-swept laser and an optical power meter, as we introduced above. The wavelength selection is achieved through Finisar WaveShaper 4000S and 16000A.

### Evaluation of the computing performance

Computing speed. As common standards in quantifying the computing speed of optical computing hardware are yet available, we follow our prior method that our OCSP’s computing speed is determined by the data rate of the output signal and the needed number of operations to yield each output symbol. Each output symbol is the dot product between the input data, within a convolution window or receptive field (determined by the scale of the chip, i.e., number of spatial paths *N*), and the weights (implemented by the MZIs and PSs). Consequently, each output symbol is the result of *N* multiply-and-accumulate (MAC) operations. For real-valued input data (*X*_*R*_) and weights (*W*_*R*_), 2 operations (1 multiplication and 1 accumulation) are needed for each MAC operation, which leads to a computing speed of 2 *N*/Δ*T* OPS per wavelength channel. With *N* = 8 parallel spatial paths and an input data rate of 1/Δ*T* = 50 GBaud. the OCSP chip demonstrates a peak computing speed of 2 × 8 × 50 G = 0.8 TOPS per wavelength and thus 5 × 0.8 TOPS = 4 TOPS with the five parallel wavelengths.

Energy consumption. The consumed energy of the OCSP mainly comes from the PSs that manipulate light interference. In the experiment, we used standard metal heaters on SOI to control the effective refractive indices and thus phase shifts based on the thermo-optic effect in silicon. 16 heaters were used in total, each taking <40 mW to achieve 2π phase shift. We estimate the average power consumption of the heaters as 20 mW and the total energy consumption as 20 × 16 = 320 mW, resulting in an energy efficiency of: 0.8TOPS/320 mW = 2.5 TOPS w^−1^ for single-wavelength real-valued case; and 4TOPS/320 mW = 12.5 TOPS w^−1^ for five-wavelength real-valued cases. This power consumption can be further reduced with advanced thin film Lithium Niobate (TFLN) platforms that cost ultra-low, if not negligible in contrast to silicon, bias power the phase shifters^49,50^. We note that TFLN’s high bandwidth can also significantly boost the tuning/training speed of the OCSP to tens of GHz—comparable to, if not faster than, state-of-art digital electronics (for more details and comparisons of other structures, see Supplementary Note 6).

## Supplementary information

Supplementary Information for Microcomb-enabled parallel self- calibration optical convolution streaming processor

## Data Availability

All data is available in the main text or the supplementary materials.

## References

[1] Sun, J. W. et al. AI-driven projection tomography with multicore fibre-optic cell rotation. *Nat. Commun.* **15**, 147 (2024). 38167247 10.1038/s41467-023-44280-1 PMC10762230

[2] Yao, P. et al. Fully hardware-implemented memristor convolutional neural network. *Nature* **577**, 641–646 (2020). 31996818 10.1038/s41586-020-1942-4

[3] Lawrence, S. et al. Face recognition: a convolutional neural-network approach. *IEEE Trans. Neural Netw.* **8**, 98–113 (1997). 18255614 10.1109/72.554195

[4] Coughlin, T. 175 Zettabytes by 2025. https://www.forbes.com/sites/tomcoughlin/2018/11/27/175-zettabytes-by-2025/ (2018).

[5] Chopra, R. Looking beyond 400G-A system vendor perspective. https://ethernetalliance.org/blog/2021/02/24/looking-beyond-400g-a-system-vendor-perspective/ (2021).

[6] Zhou, X., Urata, R. & Liu, H. Beyond 1 Tb/s intra-data center interconnect technology: IM-DD OR coherent? *J. Lightwave Technol.* **38**, 475–484 (2020).

[7] Berikaa, E. et al. TFLN MZMs and next-Gen DACs: enabling beyond 400 Gbps IMDD O-band and C-band transmission. *IEEE Photonics Technol. Lett.* **35**, 850–853 (2023).

[8] Yang, K. Y. et al. Bridging ultrahigh-*Q* devices and photonic circuits. *Nat. Photonics* **12**, 297–302 (2018).

[9] Brasch, V. et al. Photonic chip-based optical frequency comb using soliton Cherenkov radiation. *Science* **351**, 357–360 (2016). 26721682 10.1126/science.aad4811

[10] Liu, Y. B. et al. Parallel wavelength-division-multiplexed signal transmission and dispersion compensation enabled by soliton microcombs and microrings. *Nat. Commun.* **15**, 3645 (2024). 38684690 10.1038/s41467-024-47904-2 PMC11058204

[11] Marin-Palomo, P. et al. Microresonator-based solitons for massively parallel coherent optical communications. *Nature* **546**, 274–279 (2017). 28593968 10.1038/nature22387

[12] Corcoran, B. et al. Ultra-dense optical data transmission over standard fibre with a single chip source. *Nat. Commun.* **11**, 2568 (2020). 32444605 10.1038/s41467-020-16265-x PMC7244755

[13] Jørgensen, A. A. et al. Petabit-per-second data transmission using a chip-scale microcomb ring resonator source. *Nat. Photonics* **16**, 798–802 (2022).

[14] Wetzstein, G. et al. Inference in artificial intelligence with deep optics and photonics. *Nature* **588**, 39–47 (2020). 33268862 10.1038/s41586-020-2973-6

[15] Shastri, B. J. et al. Photonics for artificial intelligence and neuromorphic computing. *Nat. Photonics* **15**, 102–114 (2021).

[16] Shen, Y. C. et al. Deep learning with coherent nanophotonic circuits. *Nat. Photonics* **11**, 441–446 (2017).

[17] Feldmann, J. et al. Parallel convolutional processing using an integrated photonic tensor core. *Nature* **589**, 52–58 (2021). 33408373 10.1038/s41586-020-03070-1

[18] Feldmann, J. et al. All-optical spiking neurosynaptic networks with self-learning capabilities. *Nature* **569**, 208–214 (2019). 31068721 10.1038/s41586-019-1157-8 PMC6522354

[19] Dong, B. W. et al. Higher-dimensional processing using a photonic tensor core with continuous-time data. *Nat. Photonics* **17**, 1080–1088 (2023).

[20] Ashtiani, F., Geers, A. J. & Aflatouni, F. An on-chip photonic deep neural network for image classification. *Nature* **606**, 501–506 (2022). 35650432 10.1038/s41586-022-04714-0

[21] Lin, X. et al. All-optical machine learning using diffractive deep neural networks. *Science* **361**, 1004–1008 (2018). 30049787 10.1126/science.aat8084

[22] Zhou, T. K. et al. Large-scale neuromorphic optoelectronic computing with a reconfigurable diffractive processing unit. *Nat. Photonics* **15**, 367–373 (2021).

[23] Chen, Y. T. et al. All-analog photoelectronic chip for high-speed vision tasks. *Nature* **623**, 48–57 (2023). 37880362 10.1038/s41586-023-06558-8 PMC10620079

[24] Brunner, D. et al. Parallel photonic information processing at gigabyte per second data rates using transient states. *Nat. Commun.* **4**, 1364 (2013). 23322052 10.1038/ncomms2368 PMC3562454

[25] Xu, X. Y. et al. 11 TOPS photonic convolutional accelerator for optical neural networks. *Nature* **589**, 44–51 (2021). 33408378 10.1038/s41586-020-03063-0

[26] Huang, C. R. et al. A silicon photonic–electronic neural network for fibre nonlinearity compensation. *Nat. Electron.* **4**, 837–844 (2021).

[27] Zhu, H. H. et al. Space-efficient optical computing with an integrated chip diffractive neural network. *Nat. Commun.* **13**, 1044 (2022). 35210432 10.1038/s41467-022-28702-0 PMC8873412

[28] Bai, B. W. et al. Microcomb-based integrated photonic processing unit. *Nat. Commun.* **14**, 66 (2023). 36604409 10.1038/s41467-022-35506-9 PMC9814295

[29] Xu, S. F. et al. High-order tensor flow processing using integrated photonic circuits. *Nat. Commun.* **13**, 7970 (2022). 36577748 10.1038/s41467-022-35723-2 PMC9797566

[30] Padmaraju, K. et al. Wavelength locking and thermally stabilizing microring resonators using dithering signals. *J. Lightwave Technol.* **32**, 505–512 (2014).

[31] Tait, A. N. et al. Feedback control for microring weight banks. *Opt. Express* **26**, 26422–26443 (2018). 30469730 10.1364/OE.26.026422

[32] Wang, T. Y. et al. An optical neural network using less than 1 photon per multiplication. *Nat. Commun.* **13**, 123 (2022). 35013286 10.1038/s41467-021-27774-8 PMC8748769

[33] Meng, X. Y. et al. Compact optical convolution processing unit based on multimode interference. *Nat. Commun.* **14**, 3000 (2023). 37225707 10.1038/s41467-023-38786-x PMC10209208

[34] Fu, T. Z. et al. Photonic machine learning with on-chip diffractive optics. *Nat. Commun.* **14**, 70 (2023). 36604423 10.1038/s41467-022-35772-7 PMC9814266

[35] Goi, E. et al. Nanoprinted high-neuron-density optical linear perceptrons performing near-infrared inference on a CMOS chip. *Light Sci. Appl.* **10**, 40 (2021). 33654061 10.1038/s41377-021-00483-z PMC7925536

[36] Wu, C. M. et al. Programmable phase-change metasurfaces on waveguides for multimode photonic convolutional neural network. *Nat. Commun.* **12**, 96 (2021). 33398011 10.1038/s41467-020-20365-z PMC7782756

[37] Gu, W. T. et al. All-optical complex-valued convolution based on four-wave mixing. *Optica* **11**, 64–72 (2024).

[38] Liu, C. et al. A programmable diffractive deep neural network based on a digital-coding metasurface array. *Nat. Electron.* **5**, 113–122 (2022).

[39] Zhang, H. et al. An optical neural chip for implementing complex-valued neural network. *Nat. Commun.* **12**, 457 (2021). 33469031 10.1038/s41467-020-20719-7 PMC7815828

[40] Zhong, C. Y. et al. Graphene/silicon heterojunction for reconfigurable phase-relevant activation function in coherent optical neural networks. *Nat. Commun.* **14**, 6939 (2023). 37907477 10.1038/s41467-023-42116-6 PMC10618201

[41] Li, K. et al. An integrated CMOS–silicon photonics transmitter with a 112 gigabaud transmission and picojoule per bit energy efficiency. *Nat. Electron.* **6**, 910–921 (2023).

[42] Xu, X. Y. et al. Self-calibrating programmable photonic integrated circuits. *Nat. Photonics* **16**, 595–602 (2022).

[43] Wang, J. J. et al. Robust characterization of photonic integrated circuits. *Laser Photonics Rev.* **19**, 2400942 (2025).

[44] Krizhevsky, A. *Learning Multiple Layers of Features from Tiny Images* (University of Toronto, 2009).

[45] Chrabaszcz, P., Loshchilov, I. & Hutter, F. A downsampled variant of ImageNet as an alternative to the CIFAR datasets. Print at https://arxiv.org/abs/1707.08819 (2017).

[46] Xiao, H., Rasul, K. & Vollgraf, R. Fashion-MNIST: a novel image dataset for benchmarking machine learning algorithms. Print at https://arxiv.org/abs/1708.07747 (2017).

[47] LeCun, Y. et al. Gradient-based learning applied to document recognition. *Proc. IEEE* **86**, 2278–2324 (1998).

[48] Chen, M. et al. Inter-symbol differential detection-enabled sampling frequency offset compensation for DDO-OFDM. *IEEE Photonics Technol. Lett.* **30**, 2095–2098 (2018).

[49] Wang, C. et al. Integrated lithium niobate electro-optic modulators operating at CMOS-compatible voltages. *Nature* **562**, 101–104 (2018). 30250251 10.1038/s41586-018-0551-y

[50] Boes, A. et al. Lithium niobate photonics: unlocking the electromagnetic spectrum. *Science* **379**, eabj4396 (2023). 36603073 10.1126/science.abj4396

