# Supplementary material for: Microcomb-enabled parallel self- calibration optical convolution streaming processor
Source: Light Sci Appl. 2026 Mar 5;15:149. doi: 10.1038/s41377-025-02093-5 (PMC12963370; doi:10.1038/s41377-025-02093-5)
Supplement: Supplementary file 1 — Supplementary Information for Microcomb-enabled parallel self- calibration optical convolution streaming processor [file 41377_2025_2093_MOESM1_ESM.pdf]

Supplementary Information for

Microcomb-enabled parallel self- calibration optical convolution streaming processor

Jiajia Wang,[1,#] Xingyuan Xu,[1,*,#] Xiaotian Zhu,[2,#] Yifu Xu,[1] Shifan Chen,[1] Haoran Zhang,[1] Yixuan Zheng,[1] Shuying Li,[1] Yunping Bai,[1] Zhihui Liu,[1] Roberto Morandotti,[3] Brent E. Little,[4] Sai T. Chu,[2] Arthur J. Lowery,[5] David J. Moss,[6] and Kun Xu[1,*]

[1]State Key Laboratory of Information Photonics and Optical Communications, Beijing University of Posts and Telecommunications, Beijing, China

[2]Department of Physics, City University of Hong Kong, Tat Chee Avenue, Hong Kong, China

[3]INRS-Énergie, Matériaux et Télécommunications, 1650 Boulevard Lionel-Boulet, Varennes, Québec, J3X 1S2, Canada

[4]QXP Technology Inc., Xi'an, China., Xi'an 710119, China

[5]Electro-Photonics Laboratory, Department of Electrical and Computer Systems Engineering, Monash University, Clayton, VIC 3800, Australia

[6]Optical Sciences Centre, Swinburne University of Technology, Hawthorn, VIC 3122, Australia

*Corresponding author: xingyuanxu@bupt.edu.cn; xukun@bupt.edu.cn

#These authors contributed equally to this work.

Supplementary Note 1: The direct-detection of optical convolutional streaming

The optical convolutional streaming processor (OCSP) processes the optical signals mapping the ideal convolution output to the optical field variation (amplitude variation for real output, amplitude and phase variation for complex output). It might introduce an error if the conversion from an optical convolutional stream to an electrical convolutional stream depends on direct detection. To quantitatively analyze the impact of conversion error, we simulated the Signal-to-noise ratio (SNR) and Mean Squared Error (MSE) with varying bias phases and the sum of taps. (discuss as follows)

The optical carrier is modulated by a lithium niobate intensity modulator (half wave voltage~6V) to yield an amplitude modulation signal. The arbitrary waveform generator (AWG, Keysight M8196A, 92 GSa/s, 32 GHz) is employed to produce the input RF signals (Signal peak-to-peak value~500mv). The RF signals from the AWG are routed to the electro-optical modulator (EOM).

The output amplitude modulation signal of EOM can be given as[S1]:

$$E_{EOM}(t) = E_{in}\cos\left(\pi\frac{v(t)}{v_\pi} + \varphi_b\right) = \cos\left(\pi\frac{v(t)}{v_\pi} + \varphi_b\right)e^{j\omega_c t} = cos(\varphi(t) + \varphi_b)\, e^{j\omega_c t}$$

Where ω_c is the frequency of the input optical field, φ_b is the bias phase, $v(t)$ indicates RF signals, $\varphi(t)$ denotes the phase change introduced by RF signals. The above equation is expanded into a power series as (with modulating small signals—max($|\Delta\varphi(t)|$)~0.26 rad in this work, the influence of higher-order terms can be ignored, omitted here for orders 6 or higher):

$$E_{EOM}(t) = e^{j\omega_c t}\left(cos\varphi_b - sin\varphi_b\varphi(t) - cos\varphi_b\frac{(\varphi(t))^2}{2!} + sin\varphi_b\frac{(\varphi(t))^3}{3!} + cos\varphi_b\frac{(\varphi(t))^4}{4!} - sin\varphi_b\frac{(\varphi(t))^5}{5!}\right)$$

The output optical information stream after the OCSP:

$$\begin{aligned} E_{OCSP}(t) &= e^{j\omega_c t}\sum h(n)\cos\left(\varphi(t-nT)+\varphi_b\right) e^{-jn\omega_c T} \\ &\cong e^{j\omega_c t}\Big(cos\varphi_b\sum h(n)e^{-jn\omega_c T} \\ &- sin\varphi_b\sum h(n)\varphi(t-nT)e^{-jn\omega_c T} \\ &- \frac{cos\varphi_b}{2!}\sum h(n)\,(\varphi(t-nT))^2 e^{-jn\omega_c T} \\ &+ \frac{sin\varphi_b}{3!}\sum h(n)\,(\varphi(t-nT))^3 e^{-jn\omega_c T} \\ &+ \frac{cos\varphi_b}{4!}\sum h(n)\,(\varphi(t-nT))^4 e^{-jn\omega_c T} \\ &- \frac{sin\varphi_b}{5!}\sum h(n)\,(\varphi(t-nT))^5 e^{-jn\omega_c T}\Big) \end{aligned}$$

T denotes the delay step of OCSP, $h(n)$ denotes the tap coefficient (could be any real number in the experimental setup). For ω_c is an integer multiple of the OCSP's FSR, $n\omega_c T = 2n\pi$, $e^{-jn\omega_c T} = 1$. Attributed to the self-calibrating algorithm success in accurate phase control and alignment, in the above equation, the complex coefficients of each order are in phase, and only the real coefficients in front of each order factor need to be considered when detected by a photodetector (as list in Table S1).

The electrical convolutional stream depends on direct detection and can be regarded as the superposition of mutual interference among factors (Fig. S1a). We note that the interference between the zero-order factor and the first-order factor is the signal or ideal optical convolutional stream output ($\sum h(n)\,\varphi(t-nT)$, the delay and weight of input RF signal) and the interference among other orders factors is the noise, defining the SNR which is the ratio between the power of signal and noise:

$$SNR = \frac{Power\_signal}{Power\_noise}$$

To quantitatively analyze the convolution error, the Mean Squared Error between the output of the photodetector and the ideal is defined as (It should note that, before calculating MSE, the $ideal\_out$ and PD_out should be scaled to the same range):

$$PD\_out = \left(\sum h(n)\cos(\varphi(t-nT)+\varphi_b)\right)^2$$

$$ideal\_out = \sum h(n)\,\varphi(t-nT)$$

$$MSE = \frac{1}{N}(Ideal\_out - PD\_out)^2$$

We randomly generated a set of taps, and the sum of taps was calculated ($\sum\frac{h(n)}{\max(|h(n)|)} =$ -2.48). Fig. S1b shows the SNR with varying bias phases at different phase change peak-to-peak values (max ($|\Delta\varphi(t)|$)~ 0.2-0.3). Fig. S1c shows

the MSE with varying bias phases. In the region where the bias phase remains valid (blue shaded, Fig. S1b, c), the SNR is higher than 15 dB and the MSE is lower than 0.1. In practical implementations, we set the bias phase as $\frac{3\pi}{4}$, which corresponds to the orthogonal bias point for the intensity modulation.

To evaluate the impact of tap coefficients, we fixed the bias phase as $\frac{3\pi}{4}$ and the tunning phase peak-to-peak values as 0.26 rad, then randomly generated 5000 sets of 8-tap filters. As shown in Fig. S1 d,e, which corresponds to the SNR and MSE with varying the sum of tap coefficients, when the absolute value of tap sum is lower than 2 (for 8-tap filters as used in this work), the SNR and MSE decline sharply. Although we break it down into two sets of taps for the sum of taps that didn't satisfy this condition in this experiment, the output of OCSP could be detected using coherent methods to realize directly the conversion from an optical convolutional stream to an electrical convolutional stream.

Table | S1 Expansion of OCSP output optical field

Order number	Coefficient	Factor
Zero	$cos\varphi_b$	$\sum h(n)$
First	$-sin\varphi_b$	$\sum h(n)\varphi(t-nT)$
Second	$-\frac{cos\varphi_b}{2!}$	$\sum h(n)(\varphi(t-nT))^2$
Three	$\frac{sin\varphi_b}{3!}$	$\sum h(n)(\varphi(t-nT))^3$
Four	$\frac{cos\varphi_b}{4!}$	$\sum h(n)(\varphi(t-nT))^4$
Five	$-\frac{sin\varphi_b}{5!}$	$\sum h(n)(\varphi(t-nT))^5$

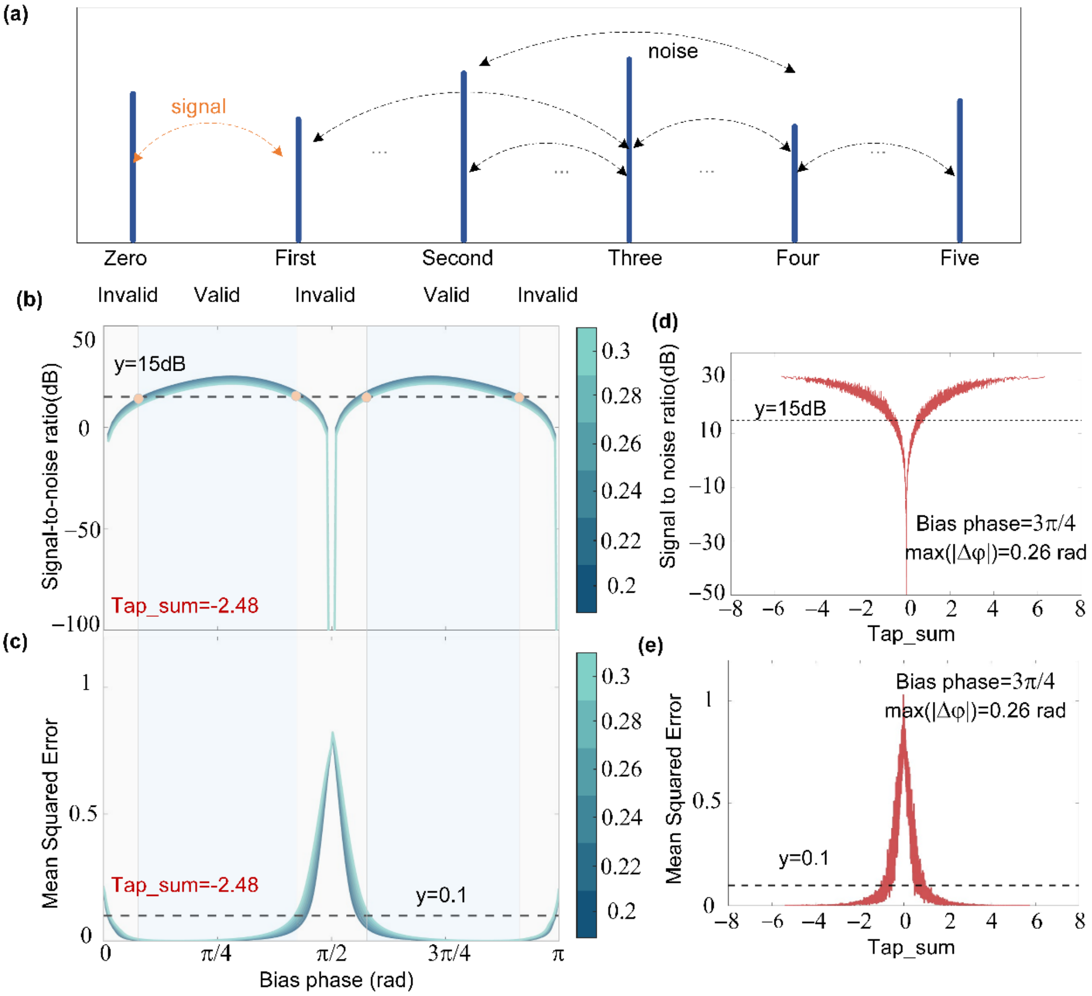

Figure S1 | Error analysis with direct detection. (a) Conceptual diagram of direct detection, the output originates from internal interferences among factors. (b) Simulated signal-to-noise ratio with varied bias phases, with different colors indicating distinct peak-to-peak values of the phase change (c) Simulated Mean Squared Error with varied bias phase. (d) Simulated signal-to-noise ratio with varied tap coefficient and. (e) Simulated Mean Squared Error with varied tap coefficient.

Supplementary Note 2: The impact of phase shift errors on convolutional computation accuracy

In practical implementations, environmental factors such as thermal drift can introduce phase errors in the taps. Therefore, it is essential to systematically evaluate the impact of tap phase errors on computational accuracy. The phase errors are mainly caused by two factors: (1) random phase fluctuations induced by temperature variations, and (2) linear phase shifts resulting from misalignment between the comb lines and the FIR filter's insertion loss spectrum.

Random phase fluctuations. We first conduct simulations to investigate the impact of random phase errors on the accuracy of convolution operations. In this simulation, we randomly generated 500 tap sets and applied Gaussian noise with varying intensities to each set. The mean square error (MSE) variation curves for the generated 500 tap sets across different levels of noise energy are shown in Fig. S2. The simulation results show that when the standard deviation of the phase Gaussian noise is below 0.5 (the phase

error within ±2 rad, as shown in Fig. S2), the mean square error approaches zero. This indicates that our OCSP has a relatively good tolerance for phase errors.

Misalignment between the repetition rate of the microcomb and the FSR of the FIR

In this study, we perform parallel convolution operations by leveraging microcombs in conjunction with the periodic interference spectra of the OCSP. This requires that the repetition rate of the microcomb aligns with the FSR of the OCSP. However, due to reasons such as process errors, there may be a mismatch between the repetition frequency and the FSR. The wavelength/ frequency drift introduces a linear phase shift to each path of the OCSP. For example, when the central wavelength is at 1550 nm, the drift range within 1 pm, the resulting linear phase variation can be approximated as negligible (calculated below).

$$d\varphi = 2\pi \times n \times df \times T = -n \times \frac{C}{\lambda^2} d\lambda \times \frac{1}{FSR}$$

$$= -2\pi n \times \frac{3 \times 10^8}{(1.55 \times 10^{-6})^2} \times 1 \times 10^{-12} \times \frac{1}{50 \times 10^9} = 0.0157 \times n\, rad$$

Where *n* denotes the nth path of the OCSP.

To evaluate the impact of the mismatch between the repetition frequency and the FSR. We randomly generated 200 sets of 8-tap filters, Fig. S2b shows the variation of the mean squared error (MSE) value with the frequency drift. The simulation indicates that for an FSR of 50 GHz, when the frequency drifts within 1 GHz, the MSE remains at a relatively low level, verifying that the coherent interleaved convolutional computing architecture also shows robustness against frequency drift. For future implementations, the integration of micro-heaters to realize tunable optical delay lines presents a viable strategy to compensate for delay inaccuracies induced by fabrication variations.

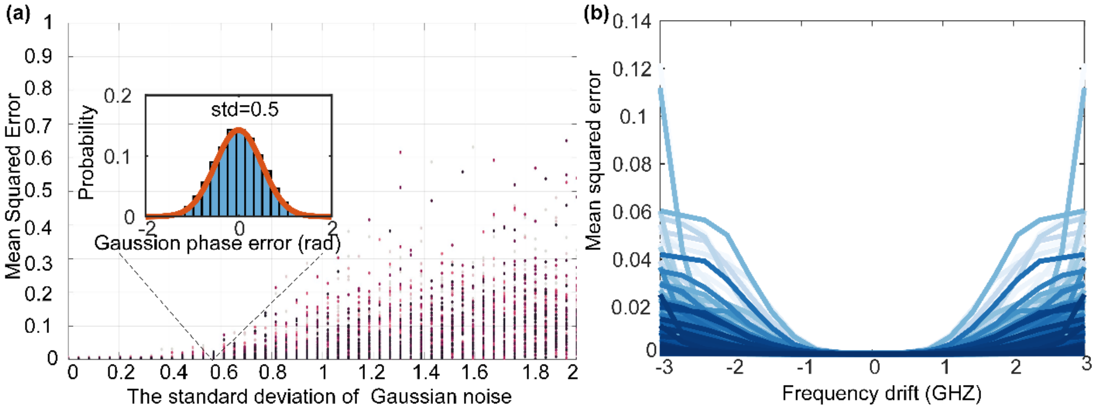

Figure S2 | (a) Simulated Mean Squared Error varied with tap coefficient and the histogram of Gaussian distribution with a standard deviation of 0.5. (b) Simulated Mean Squared Error varied with frequency shift and lines with distinct colors denote different tap coefficients.

Supplementary Note 3: The coherent detection of optical convolutional streaming

As mentioned above, the direct detection method could achieve the conversion from optical convolution flow to electrical convolution flow, where the tap setting of OCSP satisfies certain conditions, while the coherent detection method could realize the more universal conversion, eliminating the restrictions on tap coefficients (The theoretical deduction is as follows).

Take the balanced detection technology as an example, the input optical fields can be written in terms of their powers, frequencies, and phases:

$$E_{oscp}(t) = e^{j\omega_s t}\sqrt{P_s(t)}e^{j\varphi_s(t)} = e^{j\omega_s t}(\sqrt{P_s(t)}\cos(\varphi_s(t)) + j\sqrt{P_s(t)}sin(\varphi_s(t)))$$

$$E_{LO}(t) = \sqrt{A_{LO}}e^{j\varphi_{LO}}e^{j\omega_{LO}t}$$

$\sqrt{P_s(t)}$, $\varphi_s(t)$ denotes the amplitude and phase variation of the signal optical field. Assuming the Optical mixer with a perfect 50/50 power splitting and detectors with an identical responsivity R, the output photocurrents are given by [S2]:

$$I\_u(t) = \frac{R}{2}\left\{A_{LO} + P_s(t) + 2\sqrt{A_{LO}}\sqrt{P_s(t)}\cos\left((\omega_s - \omega_{LO})t + \varphi_s(t) - \varphi_{LO}\right)\right\}$$

$$I\_l(t) = \frac{R}{2}\left\{A_{LO} + P_s(t) - 2\sqrt{A_{LO}}\sqrt{P_s(t)}\cos\left((\omega_s - \omega_{LO})t + \varphi_s(t) - \varphi_{LO}\right)\right\}$$

Each photocurrent comprises three contributions. Two are proportional to the individual power of each interfering field, while the third one is proportional to an interference term dependent on the relative phase between the fields. When the fields oscillate at the same optical frequencies (homodyne detection), The photodiodes are connected to provide the differential current:

$$I(t) = 2R\sqrt{A_{LO}}\sqrt{P_s(t)}\cos\left(\varphi_s(t) - \varphi_{LO}\right)$$

For real-valued output with the phase $\varphi_s(t)$ varying between 0 and π, the terms $\sqrt{P_s(t)}sin(\varphi_s(t))$ do not convey any information. the homodyne detection method is sufficient for real-valued optical field out while the phase of local oscillator light is usually set to 0.

In the case of complex-valued output, although only getting the $\sqrt{P_s(t)}\cos(\varphi_s(t))$ terms using the homodyne detection method, all-optical vector signal information could be acquired by employing the phase diversity reception technique[S3].

We note that the OCSP is capable of performing complex value operations combined with coherent detection technology to further improve computing power and promote the application of OCSP for complex scenarios.

Supplementary Note 4: The self-calibration algorithm

The overall on-chip system consists of convolution operation units (COU) coupled in parallel with a reference path via a tunable coupler. Two pairs of on-chip ports are used: one pair accessing the whole chip for characterization and thus calibration, and the other pair accessing solely COU for feature extraction. The details of the self-calibration algorithm are introduced in Fig. S3. Compared with our previous work on the self-calibration algorithm[42], we mapped the convolutional kernel to the impulse responses of COU and improved the COU impulse response recovery algorithm to address the issue of chip noise limitation[43]. The Gap method eliminates the constraint of the Kramers-Kronig (KK) condition enabling more optical power allocation to COU. Consequently, upgraded impulse response recovery accuracies in noise-limited scenarios. Each kernel training iteration contains several steps: (1) Mapped the kernel to the desired impulse response of COU with flatting and reweighting. (2) The insertion loss spectrum of the OCSP chip was measured and the impulse response of OCSP was

recovered using the Gap method. (3) The power splitting ratios of the MZIs and phase shifts of the phase shifters (PS) were recovered from the tap coefficients of the OCSP and then compared with the desired values to yield errors, which were finally used to update the electrical power applied to the chip. the errors of the power splitting ratios of the MZIs and phase shifts of the PSs are defined as:

$$dSR_{err-MZI} = SR_{ideal} - SR_{measured}$$

$$PS_{error} = PS_{ideal} - PS_{measured}$$

SR_{ideal} denotes the ideal MZIs' splitting ratio, $SR_{measured}$ denotes the real-time measured MZI splitting ratio, PS_{ideal} denotes the ideal phase shifts of the PSs and $PS_{measured}$ denotes the measured phase shifts of the PSs. In this experiment, when $|dSR_{err-MZI}| < 0.2$, the learning rate of the corresponding MZI is set to 0. When $|PS_{error} - 2n\pi| < 0.05$, the learning rate of the corresponding phase shifter is set to 0. When the error value exceeds this range, the self-calibration algorithm is activated to adapt to the changes in the external environment. A learning rate of <1 is employed to guarantee the convergence of the learning process.

The Self-calibration algorithm offers a robust solution for accurate measurement and control of OCSP, which has been an outstanding problem so far. This algorithm effectively addresses the challenge of precise phase control and alignment in coherent optical convolution structures, thereby optimizing the utilization of spatial-division parallelism—a powerful and scalable computing paradigm.

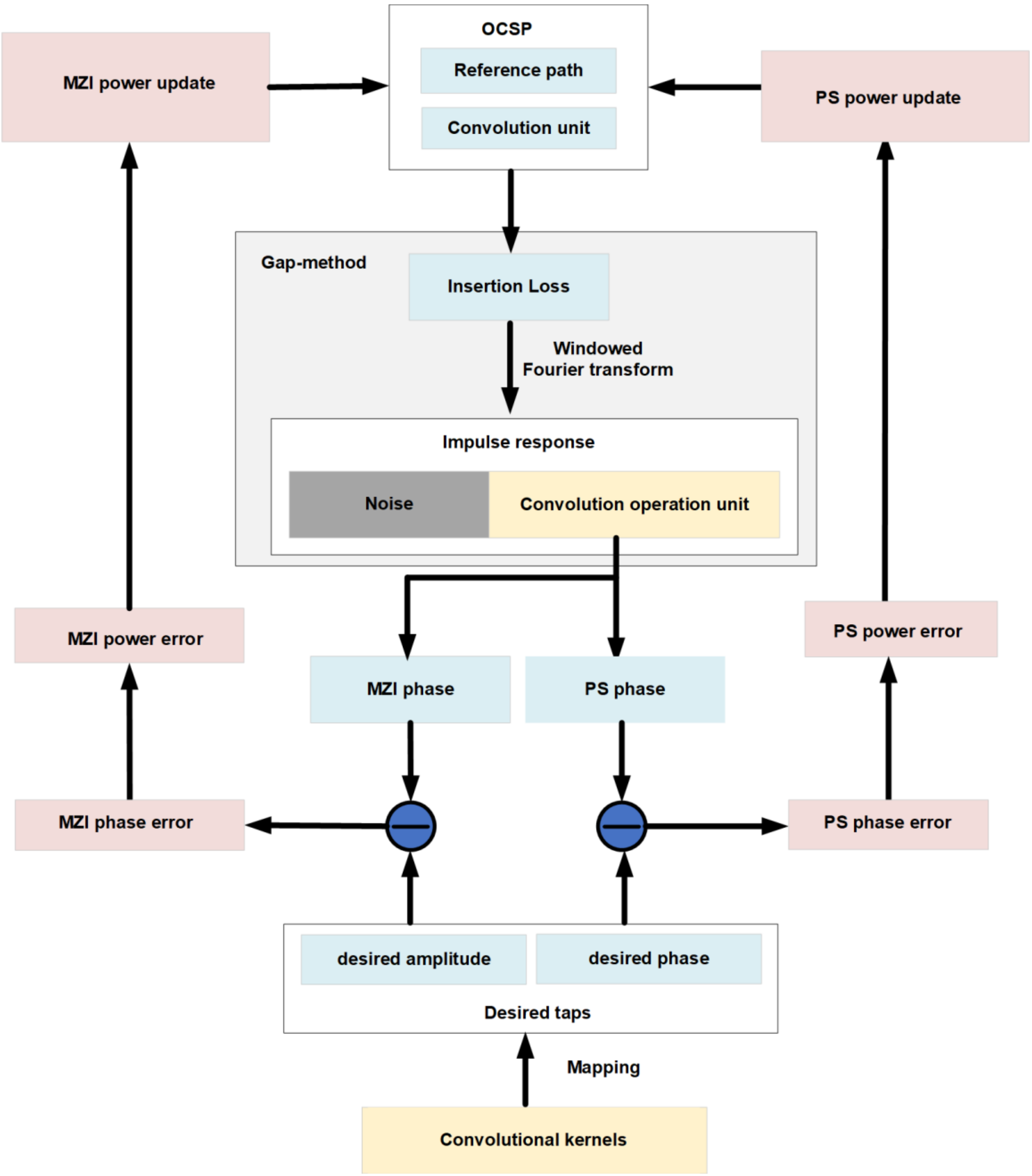

Figure S3 | Diagram of the self-calibration algorithm.

Supplementary Note 5: Robustness of the self-calibration algorithm

We quantitatively characterized the algorithm's environmental adaptability through:

(1) Long-term stability testing under static conditions (8-hour monitoring), and (2) Robustness testing against thermal perturbations. The self-calibration algorithm demonstrates real-time adaptability to environmental disturbance. In the OCSP chip, the signal processing and calibration functions are allocated to two separate port pairs—one dedicated to computational tasks, while the other monitors weight jitter. We first experimentally monitored the fluctuation of weights' phase and amplitude within 8 hours with a temperature control accuracy of 0.01 °C. Fig. S4a illustrates the variation curves of the tap's amplitude and phase, the jitter range for the tap phase is within 0.12 rad, while the jitter range for the amplitude is within 0.04 (scaled to 0-1). The measured

parameter variations demonstrate the well-maintained stability of the OCSP chip.

Furthermore, we validated the algorithm's ability to monitor and compensate for temperature-induced weight variations. The whole test process contains two steps, including:(1) Calibrate the weights to random initial values and keep the calibration algorithm running. (Fig. S4b, Area I); (2) Temperature changes and re-calibration. (Fig. S4b, Area II). In the initial step, we performed self-calibration of the OCSP weight to the target weight while maintaining a chip temperature of 30 C°. Subsequently, we kept the calibration algorithm running and monitored the changes in the weight status. In the second step, environmental temperature fluctuations ranging from -3°C to +3°C were introduced using a thermo-electric cooler (TEC) module, during which the weights of the OCSP were simultaneously recorded via the calibration port. The curves presented in area II of Fig. S4b illustrate the deterioration process of weight due to variations in environmental temperature. Such anomalies were promptly detected, triggering an automatic activation of the self-calibrating procedure that continues to operate. After several dozen iterations at a temperature of 30°C ± ΔT—distinct from the initial temperature—the weights of the OCSP were successfully restored to their initial values. The result demonstrates that the self-calibration algorithm can effectively re-calibrate OCSP weight parameters with environmental temperature variations, demonstrating its robustness and adaptability in environmental disturbance.

We note that the effectiveness/robustness of our phase recovery method under environmental disturbances is determined by whether the reference path can always remain sufficiently shorter than the streaming processor paths ($\tau > T$)[43]. As the reference path and steaming processor paths are monolithically integrated, they will suffer similar, if not identical, refractive index (and thus delay) variations upon environmental temperature fluctuations. As such, the on-chip conditions for phase recovery can always be met, guaranteeing the stability and reliability of our approach. This is further verified by experimental tests including: a) the consistency of recovery tap amplitudes (<0.04) and phases (<0.12 rad) within 8 hours (this can be further improved by optimizing the external temperature controller), as shown in Fig. S4a; b) convergence of the taps' training curves under abrupt temperature perturbations (up to ±3 °C), as shown in Fig. S4b.

By employing special packaging techniques, such as thermally expansion-matched materials, and phase change material (PCM) buffer layers, the robustness of photonic chips in varying environments can be further enhanced, ensuring that their optical performance remains unaffected by external temperature fluctuations[S4, S5]. For high-power photonic chips, traditional cooling methods may not be sufficient. Embedding microfluidic cooling channels in the packaging substrate (such as silicon interposers or ceramic substrates) and circulating deionized water or ethylene glycol solution can efficiently dissipate heat[S6].

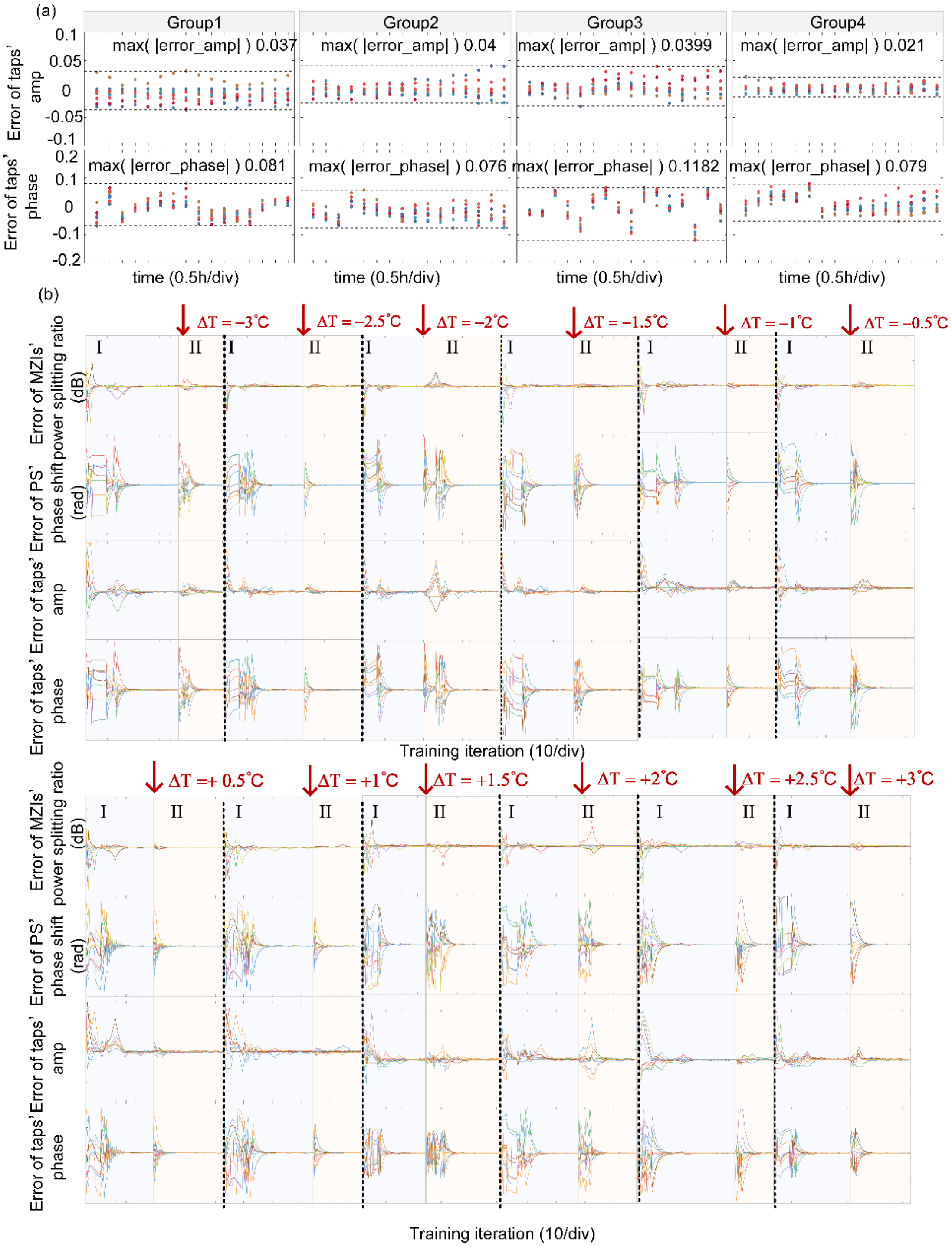

Figure S4 | Robustness testing of self-calibration algorithms. (a) Four groups of taps are randomly generated (each group consists of 8 taps, distinguished by different colors). Experimentally, the jitter of the amplitude and phase of the four groups of taps over time is measured (recorded every half an hour for 8 hours), with the temperature control accuracy of 0.01 °C. (b)The weights and phases of the taps were trained to different initial values (separated with black dotted lines), keeping the calibration algorithm running, during which disturbances of varying temperatures were added. The error curve of the MZIs' power splitting ratio (first, fifth row) and the phase shifters' phase shift (second, sixth row). The error curve of the taps' amplitude (third, seventh row) and the error curve of the taps' (fourth, e'ighth row). The taps'

amplitude and phase can reconverge to their initial states within just a few iterations after temperature perturbations, demonstrating the robustness of self-calibration algorithms.

Supplementary Note 6: Comparison of state-of-the-art photonic computing architectures

To compare the performance of various photonic computing architectures, we first systematically evaluate two key performance metrics of our OCSP system: (1) Computation density, and (2) Energy efficiency.

Photonic core compute density is defined as follows[28]:

$$compute\ density = \frac{Computing\ speed\,(TOPS)}{Area\ of\ photonic\ linear\ operations\ unit\,(mm^2)}$$

The photonic linear computing unit occupies approximately half of the total chip area, which can be roughly estimated as 10 mm^2. the photonic core compute density of our OCSP chip is 0.4Tops mm^{-2} for five-wavelength cases; and 0.08Tops mm^{-2} for single-wavelength cases.

To evaluate the energy efficiency of our OCSP system more precisely, we define the metric.

$$OCSP\ chip\ energy\ efficiency = \frac{Computing\ speed(TOPS)}{The\ power\ consumption\ of\ OCSP\ chip}$$

The energy efficiency of our OCSP chip is 2.5 TOPS W^{-1} for single-wavelength cases; and 4TOPS/320 mW=12.5 TOPS W^{-1} for five-wavelength cases. However, the complete OCSP computing architecture incorporates additional critical components to enable parallel convolution operations. It is essential to characterize the energy efficiency of the system. thereby, we define another metric:

$$Overall\ energy\ efficiency = \frac{Computing\ speed(TOPS)}{The\ power\ consumption\ of\ system}$$

The power consumption of the OCSP system is mainly from five aspects: pump-laser, erbium-doped fiber amplifier (EDFA), OCSP chip, modulator drivers, and photodetector. The waveshaper can be replaced by passive wavelength multiplexers/demultiplexers. The actual power consumption is listed in Table S2. In potential application scenarios, the OCSP chip architecture shows compatibility with optical interconnects, demonstrating the capability to process WDM signals from optical transmitters, enabling parallel computing capabilities without additional power penalties.

Table | S2 Estimated power consumption of parallel convolution system

Components		Voltage(V)	Current(A)	Power(w)
WDM signal generation	Pump-laser	11	1	11
	EDFA	5	3	15
	Modulator drivers	12	0.05	0.6
Photodetector		N/A	N/A	0.7

TEC for Microcomb<br>TEC for OCSP chip	0.2	0.2	0.04×2
OCSP chip	N/A	N/A	0.32
Total power consumption	27.7 W		
Overall energy efficiency	0.144TOPS W^{-1}		

The key performance metrics of state-of-the-art integrated photonic computing units are quantitatively compared in Table S3 (the energy efficiency and compute density metrics in Table S3 specifically pertain to the photonic computation unit). Thanks to the ultra-high-speed information access capability and multi-channel parallel processing capability, our OCSP chip demonstrates significant advantages in both computing speed and energy efficiency. Below, we analyzed the advantages in comparison with the techniques listed in Table S3 from the key improvements.

Scalable Computing Parallelism. This approach decouples the wavelength dimension from the on-chip components, establishing two independent scaling dimensions. By integrating wavelength division multiplexing (WDM) technology, this architecture enables highly parallelized optical convolution processing across multiple spectral channels. Thereby, overcoming the traditional scalability limitations (the trade-off between parallelism and component integration scale) inherent in microcomb-based non-coherent computing architectures[28,29,33].

Ultra-high-speed Data Loading. The non-flat frequency response of the micro-ring resonator (MRR) can result in broadband signal distortion, limiting the loading rate of the original signal. The coherent optical computing architectures based on MZI-mesh, high-speed signal loading pose challenges in synchronization among signals[39]. In contrast, within single-input single-output space-time interleaving architectures, the symbol rate is dictated by the free-spectral range (FSR) of finite impulse response (FIR) filter, which corresponds to the delay step between adjacent spatial paths. FSR of several hundred gigahertz (Symbol rates reach several hundred gigabaud) can be achieved in standard silicon-on-insulator (SOI) platforms by simply reducing the delays of the spirals, which lead to even better performances (smaller losses).

Weight Configuration. We apply the self-calibration algorithm to OCSP chip which only adds an optical reference path to the chip, enabling accurate setting of the synaptic weights and robustness to external temperature disturbances.

Performances and Potentials. By simultaneously enhancing the single-channel data loading rate and the multi-channel data parallel processing capabilities, the OCSP chip demonstrates its potential for seamless integration within ultra-high-bandwidth (THz-scale) and ultra-high-speed (hundreds of gigabits per second) photonic interconnection systems — this is beyond the capability of prior arts[25,28,29,33,39]. Meanwhile, thanks to the ultra-high-speed information access capability and multi-channel parallel processing capability, our OCSP chip demonstrates significant advantages in both computing speed and energy efficiency compared to prior arts[28,29,33,39].

Table | S3 Comparison of state-of-the-art photonic computing architectures

	Configurable compute speed (TOPS)	Energy efficiency (TOPS W^{-1})	Footprint (Tops mm^{-2})	Data loading rate (GBaud)	Weight configuration	Neural Network
This work	4	12.5	0.4	50	Self-configuration	Fashion MNIST(10) 91.71%
Microcomb[25]	11	N/A	N/A	62.5	N/A	MNIST(10) 88%
MRR array[28]	0.136	3.4	1.04	17	Look-up table + gradient-descent control method	MNIST(10) 96.6%
MRR array[29]	0.48	N/A	0.104	20	Look-up table	KTH(5) 97.9%
MRR array[33]	0.265	0.2	0.58	16.6	Look-up table	MNIST(10) 92.17%
MZI mesh[39]	N/A	N/A	N/A	1×10^{-5}	Look-up table	MNIST (10) 90.5%

Supplementary Note 7: The accuracy of parallel computing

We utilize the scatter plot to illustrate the accuracy of convolution calculations within the parallel computing system[28]. The X-axis and Y-axis of the scatter plot represent experimental data and simulation data, respectively. A distribution of points that closely aligns with the 45° diagonal line indicates a higher degree of consistency between simulation results and experimental results. The dispersion of these points serves as an indicator of error magnitude; greater dispersion corresponds to larger errors. Additionally, the accompanying error distribution histogram and Gaussian fitting curve provide insights into this error distribution. The standard deviation (σ) of Gaussian-fitted residuals quantitatively reflects the fundamental precision limit of our parallel convolution system. Fig. S5 shows the convolution operation results of three different convolution kernels at five wavelengths. Kernel1 and Kernel2 are used to verify the general image processing functions (as shown in the main text), while Kernel3 is used to test the convolution calculation accuracy near the critical point. The data points of Kernel1 and Kernel2 are closely distributed around the diagonal, and their residual distributions show the Gaussian shape ($\sigma < 0.05$).

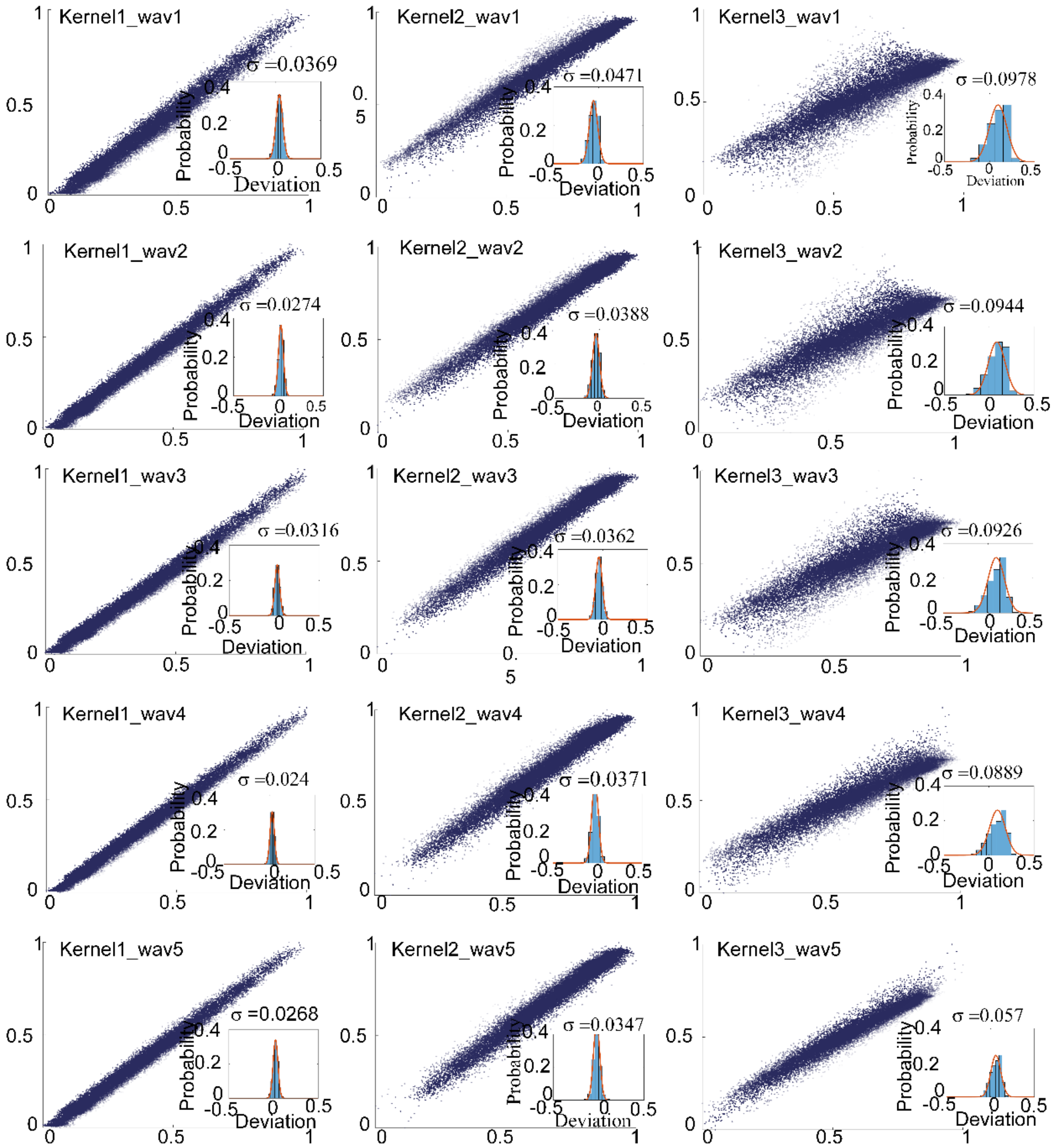

Figure S5 | The scatter plot of parallel convolutional computation. The three columns respectively represent the scatter plots of the convolution calculations of three distinct convolution kernels. Different rows in each column represent different carriers.

The experimental errors may originate from the system's noises, inter-channel crosstalks, inter-symbol interferences and photodetection introduced nonlinearity. Subsequently, we will conduct the in-depth analysis of the main error mechanisms.

System limitations. Here we analyze the impact of system limitations (noise, bandwidth, etc) on the fidelity of the original waveform. The original image data, after being shaped by a root-raised cosine filter (roll-off factor set as 1), was loaded onto a single-wavelength channel with a symbol rate of 50 GBaud, which was subsequently detected by the photodetector. Fig. S6a shows the amplitude response of the system measured via multi-tone signals. The amplitude response indicated higher loss at frequencies higher than ~20 GHz (the notch at ~25 GHz was induced by the photodetector). Fig. S6b shows the amplitude spectrum of the original signal with a

bandwidth of ~25 GHz. Fig. S6c shows the waveform received by the oscilloscope. The limited system bandwidth induces pronounced waveform distortion, particularly in regions with high-frequency components. Furthermore, the error distribution histogram reveals a certain level of discretization. When the original waveform is processed by different convolution kernels with varying frequency responses, the output waveform that retains a higher proportion of high-frequency components exhibits greater distortion due to bandwidth constraints. Fig. 3b in the manuscript illustrates the frequency responses associated with three distinct convolution kernels. Fig. S5 in the supplementary demonstrates that Kernel 1 features a superior Gaussian variance in comparison to both Kernel 2 and Kernel 3. This enhancement can be attributed to Kernel 1's frequency response, which effectively concentrates spectral energy in low-frequency components while attenuating high frequencies, thereby mitigating distortion caused by bandwidth limitations.

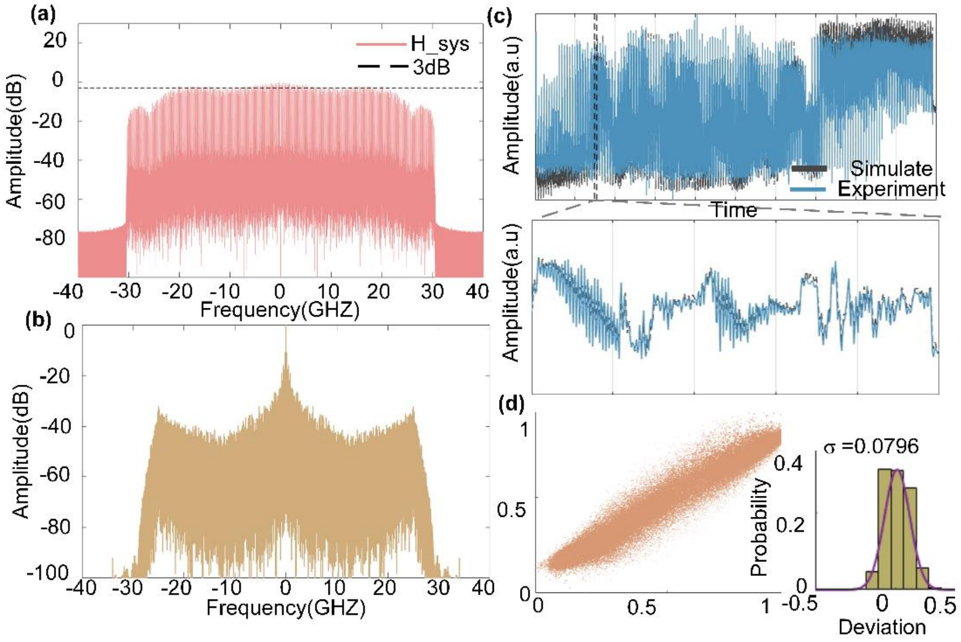

Figure S6 | Systematic error analysis. (a) The amplitude response of the system. (b) The amplitude spectrum of the original signal. (c) The original waveform captured by the oscilloscope and its zoom-in view. (d) Scatter plot distribution and error distribution histogram of the received waveform and the original transmitted waveform.

Direct detection introduced nonlinearity. The optical convolutional streaming processor (OCSP) processes the optical signals and maps the ideal convolution output to the optical field variation. In this study, direct detection is adopted to convert the calculated optical data stream into an electrical data stream. However, it introduces non-negligible errors when the signal-signal interference (SSBI) terms become non-negligible in contrast to carrier-signal interference terms, especially when the carrier is suppressed (i.e., located at the zero point of the amplitude response, see Supplementary Note 1 for detailed discussions). As shown in the frequency response corresponding to Kernel 3, the frequency response exhibits a slight suppression of the carrier. In such case, to enhance the accuracy of convolutional computations, one approach is to decompose the convolutional kernel. Additionally, the optical carrier-to-signal ratio of the original input can be appropriately increased to compensate for the optical carrier loss incurred due to the frequency response. Fig. S7a shows the MSE values varies with

carrier-first sideband ratios (by adjusting the bias point of the modulator), when this ratio surpasses 27dB, the curve tends to converge. Fig. S7b characterizes the convolution output waveform and scatter plot distribution with 30 dB carrier-first sideband ratios, where the residual error histogram (inset) exhibits near-perfect Gaussian distribution, quantitatively verifying that the sources of error is dominated by the Gaussian noises. In addition to this, the conversion of optical data streams to electrical data streams can be achieved using coherent receivers to avoid the nonlinearity introduced by the direct detection.

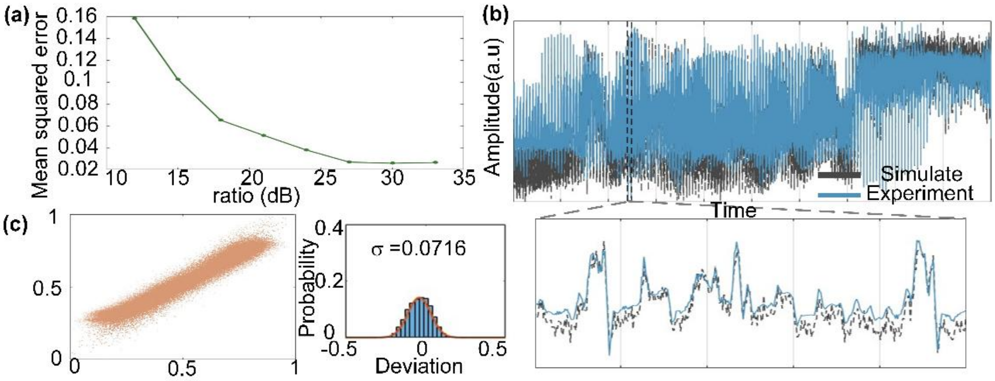

Figure S7 | Nonlinearity error analysis. (a) The MSE values varies with carrier-first sideband ratios. (b) The convolution output waveform with 30 dB carrier-first sideband ratios. The black dashed line and blue solid line are the calculated (ideal) and experimentally obtained waveform, respectively. (c) Scatter plot and residual error distribution histogram. The residual error distribution histogram shows a standard deviation of 0.0716.

Inter-channel crosstalk. Inter-channel crosstalk in WDM systems mainly arises from insufficient channel spacing, resulting in spectral overlap between adjacent wavelength channels. In this work, the comb lines' spacing is ~50 GHz, and the energy of the original signal is predominantly concentrated within the range of 25 GHz (as shown in Fig. S6b). Therefore, the error introduced by spectral aliasing between adjacent wavelength channels is negligible here. Furthermore, the channel spacing can be further increased by simply using comb lines with larger spectral intervals (i.e., every two/three comb line).

Supplementary Note 8: Collaborative design with electronic control units

Optical computing has ultra-high processing capabilities at the sub-nanosecond level, but its weight update rate is often limited by the bottleneck of the electronic control system. The clock frequency of current commercial electronic control chips is usually only at the GHz level, which is significantly lower than the tens of GHz response speed of on-chip optical modulation devices. This mismatch in speed between optics and electronics leads to idle time for photonic computing chips while waiting for weight updates, reducing the overall system efficiency. To break through this limitation, it is necessary to optimize both the hardware and algorithm levels in a coordinated manner: on the hardware side, high-speed serial interfaces such as Peripheral Component Interconnect Express (PCIe) 5.0/6.0 can be adopted to increase the bandwidth of electro-optical data interaction, while continuing to optimize the structure of electro-optical modulators based on new materials to maintain the on-chip control speed advantage of tens of GHz; on the algorithm side, an adaptive weight update mechanism can be designed to dynamically adjust the update frequency according to the real-time

state of optical computing-increasing the update frequency to match its speed advantage when optical computing is in a high-speed operation phase, and intelligently reducing the update frequency when the speed fluctuates due to complex tasks to ensure weight accuracy. This hardware-algorithm coordinated optimization approach is expected to significantly alleviate the speed mismatch problem between optics and electronics, and fully leverage the performance potential of optical computing.

Supplementary Note 9: Seamless embedding OPU in datacenter

Nowadays, the majority of data centers still employ conventional hierarchical architectures that segregate computing, storage, and networking resources into discrete layers. In this paradigm, each functional layer independently constitutes a complete computing system through the integration of fundamental components including central processing units (CPUs), memory modules, system buses, and persistent storage devices (Fig. S8a). However, this architectural inherently suffers from three fundamental limitations commonly referred to as the "three walls": the memory wall, I/O wall, and computational power wall[S7-S10]. These architectural constraints have resulted in significant bottlenecks in both data access latency and transfer bandwidth. The emerging next-generation data center computing architecture proposes a fundamental redefinition of computational infrastructure through the establishment of a data-centric, heterogeneous computing ecosystem. By implementing a unified resource pool architecture that integrates distributed computing, memory, and storage assets, the accessibility of resources at each node and the overall efficiency of the system can be enhanced simultaneously (Fig. S8b). This model of resource integration, when coupled with a high-performance, low-latency optical interconnection framework capable of adaptive topology reconfiguration, facilitates dynamic resource provisioning and optimized workload allocation to effectively address the diverse demands of various applications[S11-S13]. This architectural framework employs optical interconnects to facilitate both intra-resource-pool connectivity and inter-node communication. The OCSP can be integrated into the heterogeneous computing pool serving as a low-latency and energy-efficient optical computing unit (OPU). As Fig. S8c shows, at the WDM transmitter, each comb line carries independent data streams through WDM technology. The OCSP is directly embedded in the WDM transmission link, maintaining compatibility with existing transceiver interfaces, enabling parallel feature extraction across all wavelength channels. At the WDM receiver, the multi-channel convolutional stream is detected by the photodetector and transmitted to the next computing node after processing (such as sampling and retiming). This deployment seamlessly integrates optical computing hardware with electrical computing components through advanced optical interconnection systems, delivering hardware-level acceleration for applications such as artificial intelligence and cloud computing.

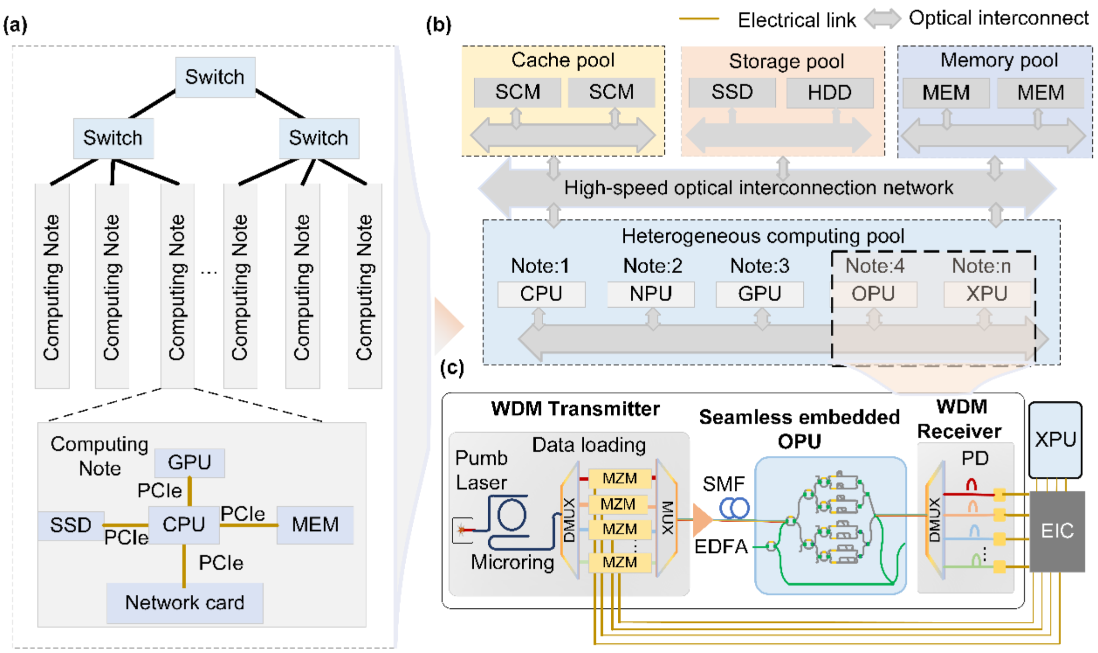

Figure S8 | The schematic diagram of the data center architecture. (a) Traditional datacenter network with hierarchical switches. SSD: Solid state drive, GPU: Graphics processing unit, CPU: Central processing unit, OPU: Optical computing unit, MEM: memory. (b) Flattened network topology with pooled resources, enabled by optical interconnect. SCM: Storage class memory, SSD: Solid state drive, NPU: Neural processing unit. (c) Schematic diagram of seamless integration of OPU into data center. EIC: Electronic integrated circuit.

The operability and stability of microcomb

This work used soliton crystal microcombs assisted by engineered mode crossings. Unlike conventional dissipative Kerr solitons requiring complex active stabilization governed by the Lugiato-Lefever equation, the microcomb exhibit: (*i*) deterministic formation through mode-interaction-mediated background waves, (*ii*) characteristic spectral fingerprints arising from dense soliton-packets interference, and (*iii*) stable operation enabled by high intracavity power. The system permits simplified initiation via adiabatic pump wavelength sweeping - achievable even through manual detuning - while showing remarkable resilience against thermal instabilities that typically plague DKS-based systems. In our previous work, we characterized the microcomb's power fluctuations over a 66-hour continuous operation period through optical spectral acquisitions at 15-minute intervals. The derived relative standard deviation of -14 dB conclusively demonstrates exceptional long-term power stability[25].

The scalability of input Information flow. Microcombs well support the scaling of parallelism, with demonstrations of up to 80 channels in the C-band at 50 GHz spacing [25, S14, S15]. The WDM transmitter generates a parallelized optical information stream by encoding data onto the micro-comb which can be achieved through the micro-ring modulators or Mach-Zehnder modulators (following the wavelengths demultiplexing). At the WDM receiver, the parallelized convolutional stream output is demultiplexed and subsequently detected by photodetectors, yielding the parallel readout of the convolutional data.

There exist numerous feasible solutions for loading data in more wavelength channels. For example, the microcomb can be first deinterleaved and then fed into different

micro-ring modulator arrays, achieving loading of 20 (demonstrated in [S16]) data streams, each at 128 Gb/s across 20 microcomb lines. The micro-ring modulators with ultra-wide free spectral range (FSR~4 THZ) [S17] have also been proposed to enable data loading across a large number of wavelength channels (4THz/50GHz = 80 wavelength channels).

The scalability of convolutional kernel. In neural networks, commonly utilized convolution kernel sizes are typically 3×3 or 5×5. For our passive OCSP structure, the size of the convolution kernel is determined by the tap coefficients of the Finite Impulse Response (FIR) filter, which corresponds to the number of delay paths. Insertion loss is a critical factor that affects the expansion capabilities of an individual convolutional kernel. For instance, a convolutional kernel with dimensions of 3×3 operating at an interface rate of 50G Baud and fabricated on a Silicon-on-Insulator (SOI) platform exhibits an insertion loss of approximately 15 dB. This performance can be further enhanced by adopting low-loss photonic integration platforms such as silicon nitride, thereby enabling the expansion of individual convolutional kernels.

Potential applications of the MRRs. As discussed above, the micro-comb serving as a multi-wavelength source and the OCSP used for parallel convolution operation, the implementation of this parallel convolutional computing system may also necessitate additional auxiliary components, such as the micro-ring modulators (MRM) to achieve parallel data loading.

Different from the application in non - coherent computing architectures, where MRRs are employed to weight different signals, the control of MRR weight stability typically relies on auxiliary detection and feedback control circuitry. Such as: the integrated monitor for obtaining MRR status information, the integrated temperature sensor for detecting the local temperature of MRR[s18]; and various novel calibration algorithms, such as the jitter signal algorithm[30, s19], the feedback control algorithm of the micro-ring weight library[31, s20], and the state locking algorithm[s21, s22] for real-time weight calibration of the micro-ring synapse.

The MRRs are mainly used for modulating signals (such as the silicon micro-ring modulator), recent advances in modulator design have shown promising performance. For instance, a silicon MRM with two-segment Z-shaped junctions demonstrated an electro-optic bandwidth of ~48.6 GHz and a modulation efficiency $V\pi \cdot L$ of ~0.6 V·cm, enabling 200 Gb/s PAM4 modulation[s23]. Another high-bandwidth Si-MRM achieved a −3 dB optical bandwidth exceeding 67 GHz and a data throughput of 300 Gbps through doping concentration optimization and optical proximity correction (OPE) technology[s24]. However, the thermal fluctuation that can cause an undesired resonance variation due to the strong thermo-optic effect present in silicon waveguides[s25]. Thus, the resonance control is necessary to insure the stability of the MRR modulator in a real-word deployment. Several methods have been reported aimed at an integrated solution for resonance control[s26-s30] (such as the non-proportional-integral-differential (PID) homodyne method). The most common approach uses a power detector and an integrated heater that can form a closed-loop via PID feedback[s31].

Supplementary Note 10: Additional experiment details and results

To verify the universality of our OCSP, we also tested its performance on different datasets including ImageNet-32, Fashion-MNIST, and MNIST. In the ImageNet-32

experiment, we randomly chose two classes from the original 1000 classes to form a subset. The training dataset for each class consisted of 1300 images, and the initial validation set 50 images. We randomly selected 50 images per class from the training set and added them to the corresponding validation sets, resulting in 100 validation images per class. We built a neural network with two convolutional layers and three fully connected layers for ImageNet-32 classification. After training, we tested the network on a total of 200 images (100 per class). The first convolutional layer (implemented on our OCSP) included three kernels, each with a $3\times2\times2$ shape. The final accuracy achieved was 86.5%, compared to 90% on electronic hardware, as shown in Fig. S10a. For the Fashion-MNIST and MNIST experiments, we trained the neural networks on their standard training sets and tested them on 350 images randomly selected from their standard testing sets. The Fashion-MNIST network contained one convolutional layer and three fully connected layers, while the MNIST network contained one convolutional layer and two fully connected layers. In both experiments, the first convolutional layers (implemented on our OCSP) included two kernels, each of 2×2 size. The accuracy on Fashion-MNIST was 84.86% (versus 90.86% on electronic hardware), and the accuracy on MNIST was 91.71% (versus 95.43% on electronic hardware), as shown in Fig. S9a. Figs. S11, S12, and S13 present example convolutional results generated by our OCSP and electronic hardware, each corresponding to input images from one of the three datasets.

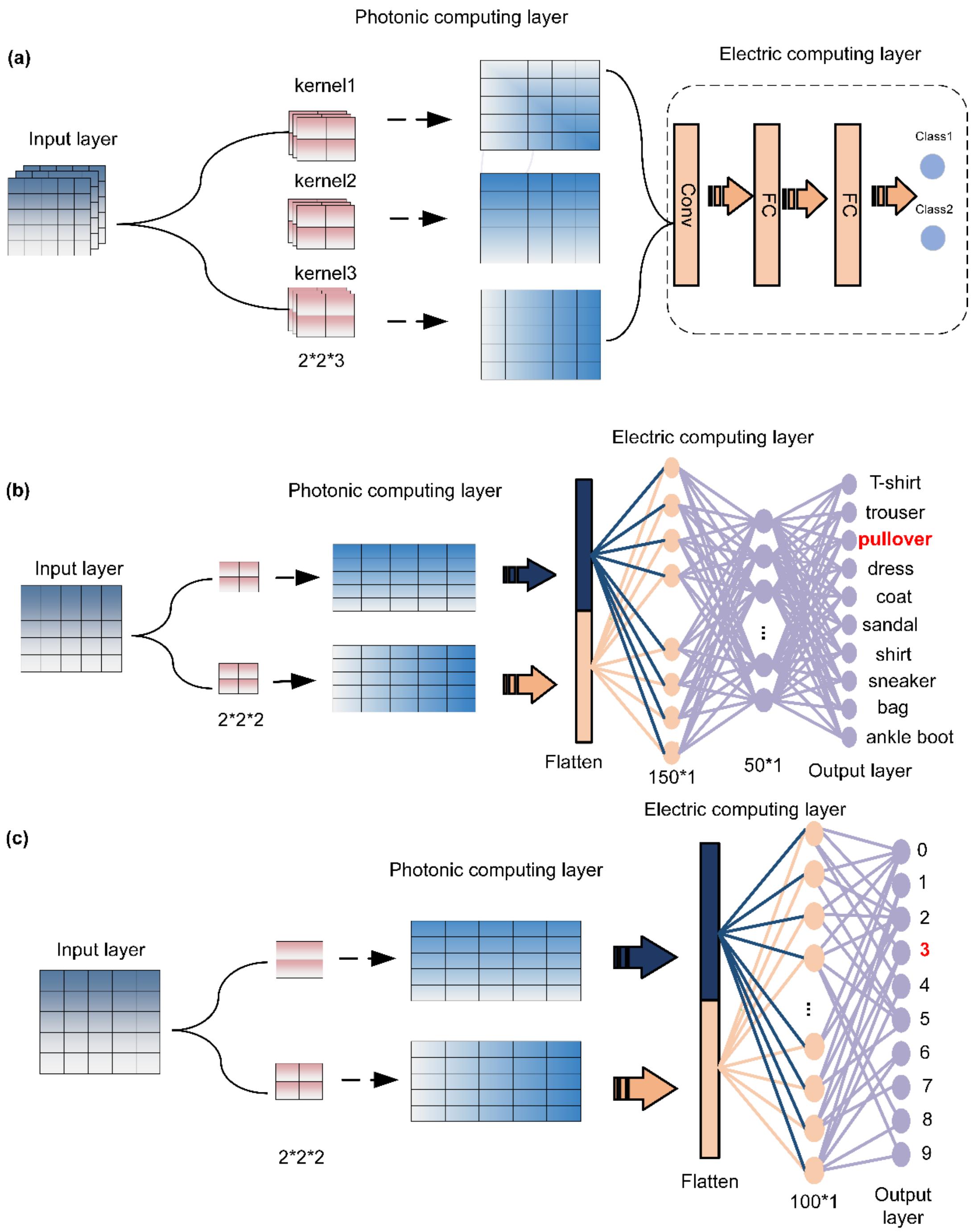

Figure S9 | Neural network architectures applied to three datasets. (a) ImageNet-32. (b) Fashion-MNIST. (c) MNIST.

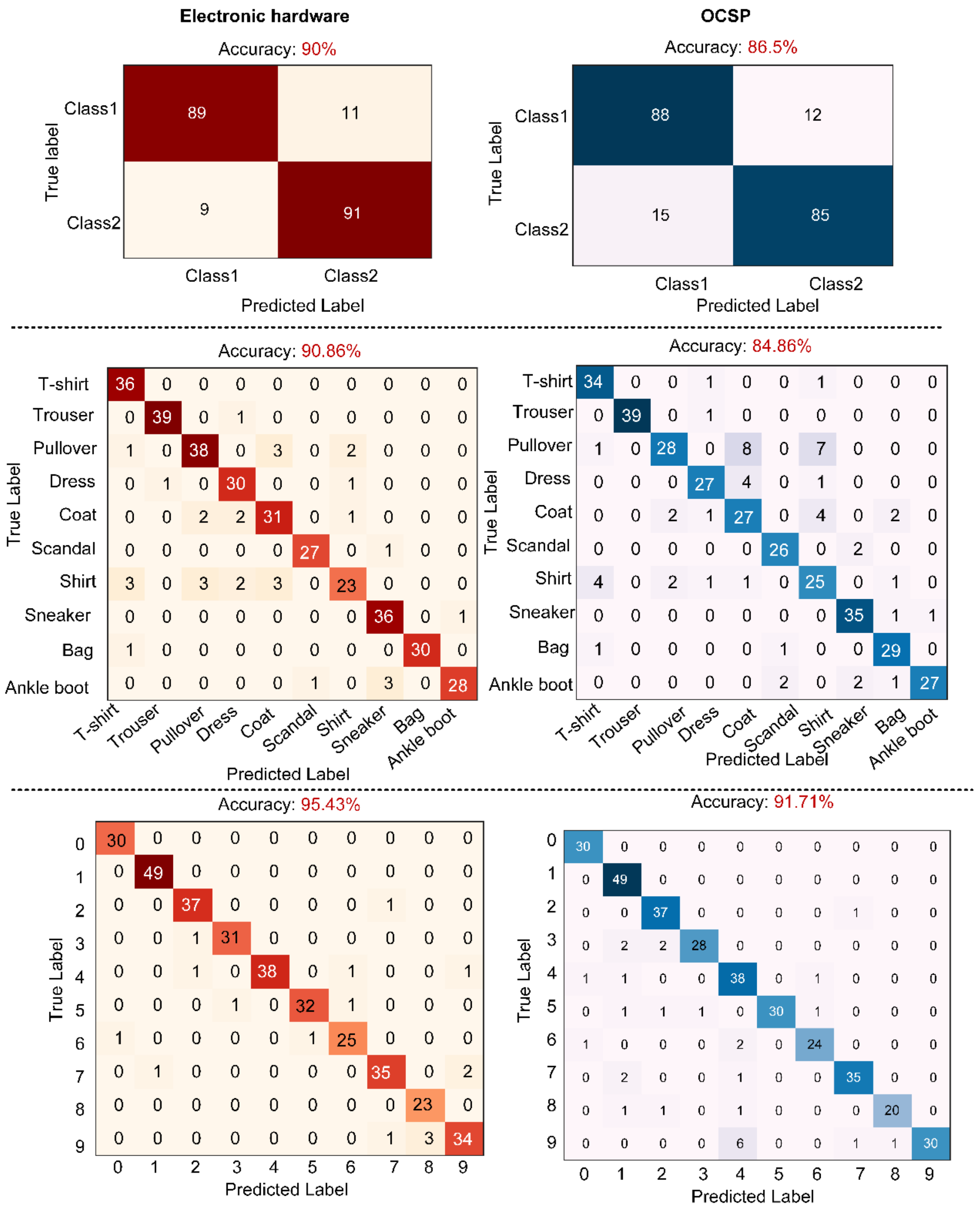

Figure S10 | Confusion matrices of classification experiments. (a) Results on ImageNet-32. (b) Results on Fashion-MNIST. (c) Results on MNIST.

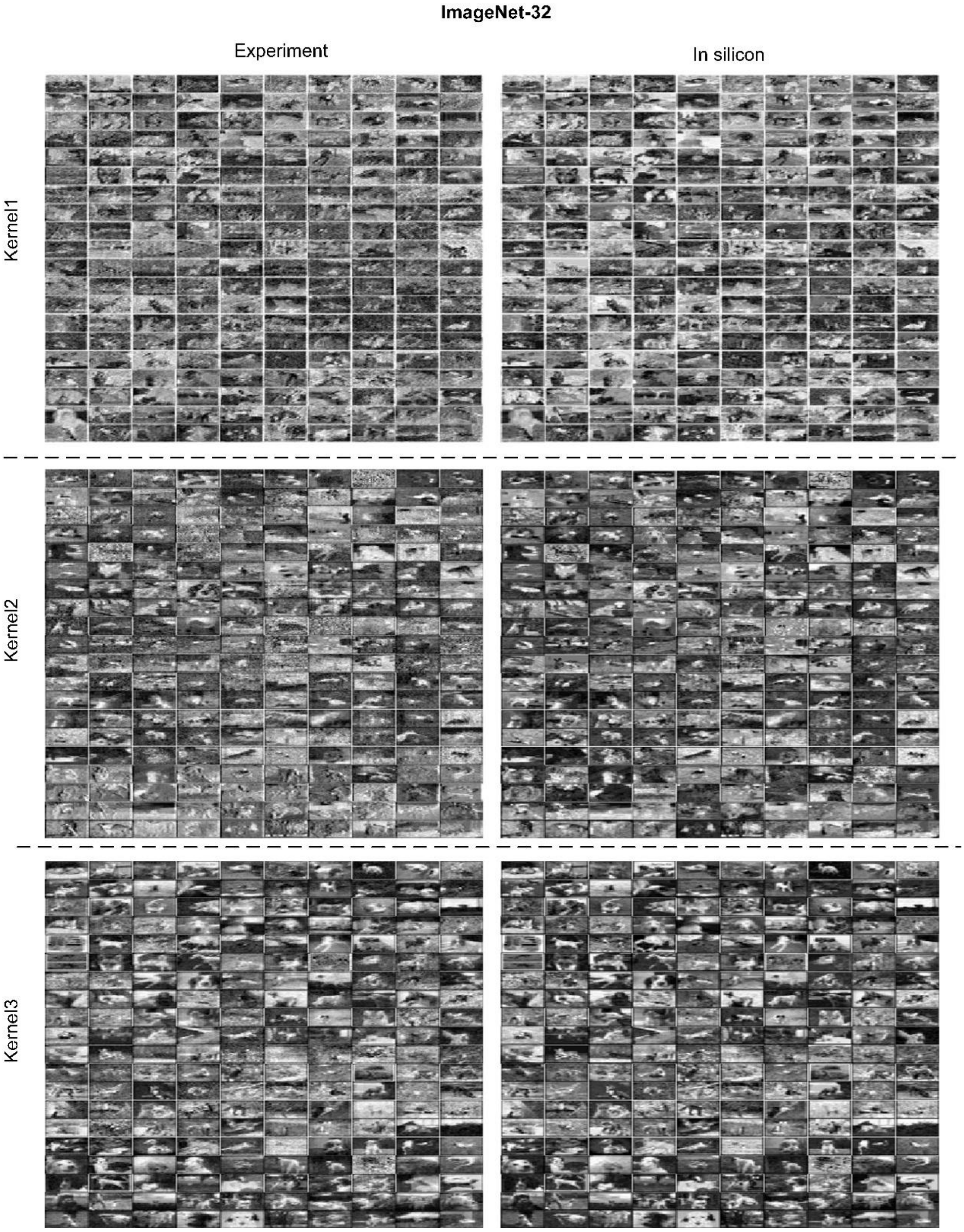

Figure S11 | Convolutional results from each kernel for input images of the ImageNet-32 dataset.

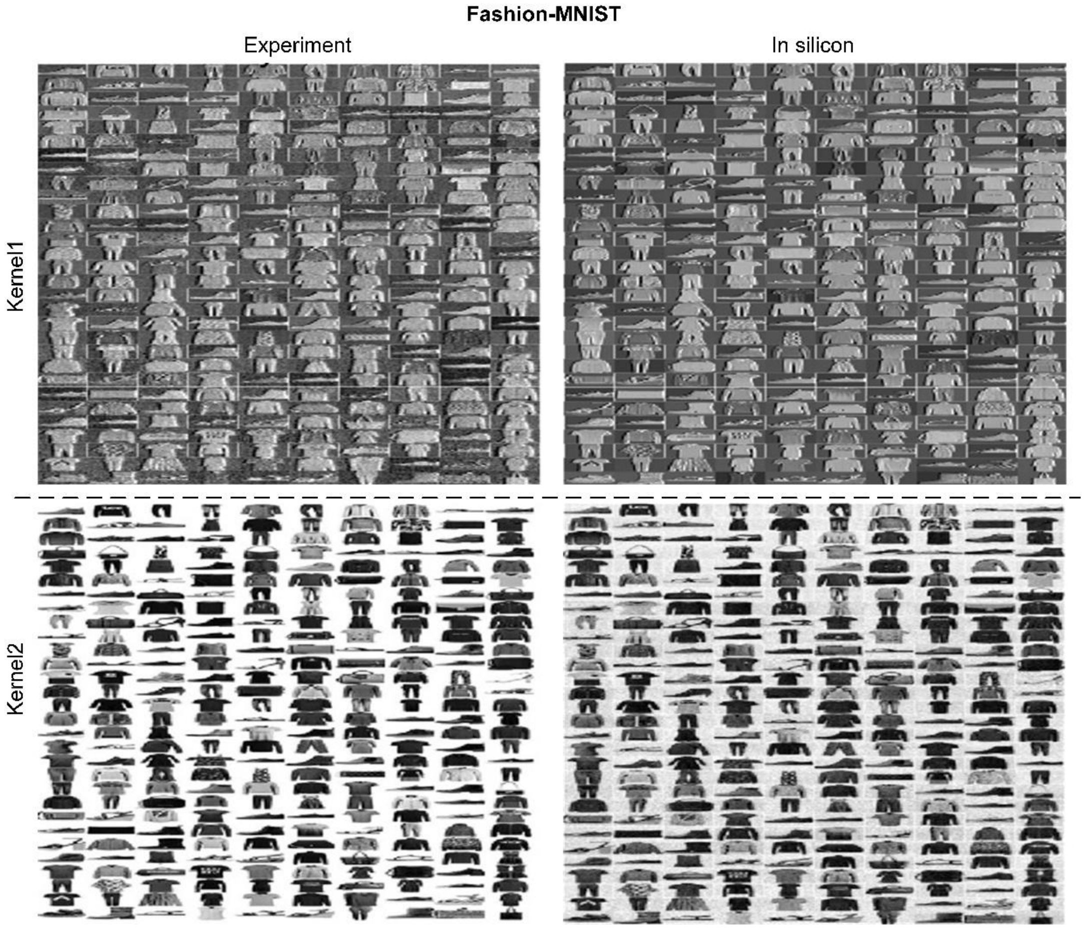

Figure S12 | Convolutional results from each kernel for input images of the Fashion-MNIST dataset

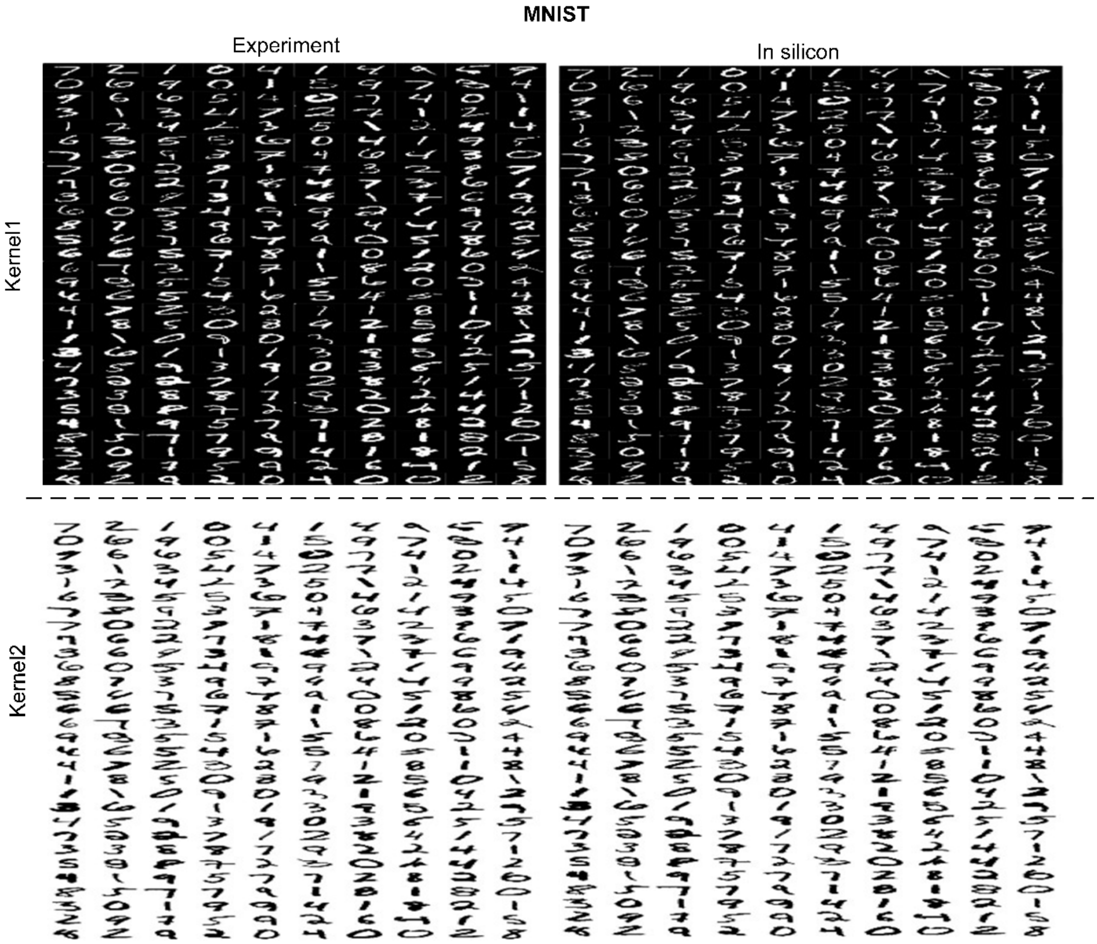

Figure S13 | Convolutional results from each kernel for input images of the MNIST dataset.

References

[S1].Mahapatra A, Murphy E J. Electrooptic modulators[M]//Optical Fiber Telecommunications IV-A. Academic Press, 2002: 258-294.
[S2].Painchaud Y, Poulin M, Morin M, et al. Performance of balanced detection in a coherent receiver[J]. Optics Express, 2009, 17(5): 3659-3672.
[S3].Fang L, Bi G, Kot A C. New method of performance analysis for diversity reception with correlated Rayleigh-fading signals[J]. IEEE transactions on vehicular technology, 2000, 49(5): 1807-1812.
[S4].Li Z, Tan J, Li J, et al. A review on thermal management of light-emitting diodes: From package-level to system-level[J]. Applied Thermal Engineering, 2024: 124145.
[S5]. Pittroff W, Erbert G, Eppich B, et al. Highly stable conductively cooled 1 kW-QCW diode laser stacks with high duty cycle[C]//2008 58th Electronic Components and Technology Conference. IEEE, 2008: 991-997.
[S6].Enright R, Lei S, Nolan K, et al. A vision for thermally integrated photonics systems[J]. Bell Labs Technical Journal, 2014, 19: 31-45.
[S7].Kevin Lim. et al. Disaggregated memory for expansion and sharing in blade servers. SIGARCH Comput. Archit. News 37, 267–278. (2009).
[S8].Michelogiannakis, G. et al. A case for intra-rack resource disaggregation in HPC. ACM Trans. Archit. Code Optim. 19, 1–26 (2022).

[S9].Levy, C. S. et al. 8-λ × 50 Gbps/λ heterogeneously integrated Si-Ph DWDM transmitter. *IEEE J. Solid-State Circuits* **59**, 690–701 (2024).

[S10]. Roytman, E. et al. AI & HPC system opportunity with integrated photonics chiplets. In *Proc. ISCA* (New York, NY, USA, Jun. 2022).

[S11]. Gonzalez, J. et al. Optically connected memory for disaggregated data centers. *J. Parallel Distrib. Comput.* **163**, 300–312 (2022).

[S12]. Zhu, Z. et al. Photonic switched optically connected memory: An approach to address memory challenges in deep learning. J. Lightwave Technol. 38, 2815–2825 (2020).

[S13]. Yoo, S. J. B. New trends in photonic switching and optical networking architectures for data centers and computing systems. J. Opt. Commun. Netw. 15, C288–C298 (2023).

[S14]. Corcoran B, Tan M, Xu X, et al. Ultra-dense optical data transmission over standard fibre with a single chip source[J]. Nature communications, 2020, 11(1): 2568.

[S15]. Xu X, Wu J, Nguyen T G, et al. Advanced RF and microwave functions based on an integrated optical frequency comb source[J]. Optics Express, 2018, 26(3): 2569-2583.

[S16]. Cai, H. et al. Microcomb and Micro-Ring Modulator Driven DWDM Optical Transmitter With Ultra-High Speed of 20 × 128 Gb/s. J. Lightwave Technol. 43, 7239-7245 (2025).

[S17]. Chan, D. W. U. et al. Ultra-Wide Free-Spectral-Range Silicon Microring Modulator for High Capacity WDM. J. Lightwave Technol. 40, 7848-7855 (2022).

[S18]. H.-T. Kim and M. Yu, "Cascaded ring resonator-based temperature sensor with simultaneously enhanced sensitivity and range," Opt. Express 24, 9501-9510 (2016).

[S19]. W. Zhang, C. Huang, H.-T. Peng, S. Bilodeau, A. Jha, E. Blow, T. F. de Lima, B. J. Shastri, and P. Prucnal, "Silicon microring synapses enable photonic deep learning beyond 9-bit precision," Optica 9, 579-584 (2022).

[S20]. C. Huang, S. Bilodeau, T. F. d. Lima, A. N. Tait, P. Y. Ma, E. C. Blow, A. Jha, H.-T. Peng, B. J. Shastri, and P. R. Prucnal, "Demonstration of scalable microring weight bank control for large-scale photonic integrated circuits," APL Photonics 5, 040803 (2020).

[S21]. A. Annoni, E. Guglielmi, M. Carminati, S. Grillanda, P. Ciccarella, G. Ferrari, M. Sorel, M. J. Strain, M. Sampietro, A. Melloni, and F. Morichetti, "Automated Routing and Control of Silicon Photonic Switch Fabrics," IEEE J. Sel. Top. Quantum Electron. 22, 169-176 (2016).

[S22]. P. Dong, R. Gatdula, K. Kim, J. H. Sinsky, A. Melikyan, Y.-K. Chen, G. de Valicourt, and J. Lee, "Simultaneous wavelength locking of microring modulator array with a single monitoring signal," Opt. Express 25, 16040-16046 (2017).

[S23]. Hu F, Zhang Y, Zhang H, et al. Beyond 300Gbps silicon microring modulator with AI acceleration[J]. arXiv preprint arXiv:2111.05331, 2021.

[S24]. Yuan, Y., Peng, Y., Sorin, W.V. et al. A 5 × 200 Gbps microring modulator silicon chip empowered by two-segment Z-shape junctions. Nat Commun 15, 918 (2024).

[S25]. K. Padmaraju and K. Bergman, "Resolving the thermal challenges for silicon microring resonator devices," Nanophotonics 3(4–5), 269–281 (2013).

[S26]. K. Padmaraju, D. F. Logan, X. Zhu, J. J. Ackert, A. P. Knights, and K. Bergman, "Integrated thermal stabilization of a microring modulator," Opt. Express 21(12), 14342–14350 (2013).

[S27]. H. Jayatilleka, K. Murray, M. Á. Guillén-Torres, M. Caverley, R. Hu, N. A. F. Jaeger, L. Chrostowski, and S. Shekhar, "Wavelength tuning and stabilization of microring-based filters using silicon in-resonator photoconductive heaters," Opt. Express 23(19), 25084–25097 (2015).

[S28]. C. Qiu, J. Shu, Z. Li, X. Zhang, and Q. Xu, "Wavelength tracking with thermally controlled silicon resonators," Opt. Express 19(6), 5143–5148 (2011).

[S29]. Y. Zhang, Y. Li, S. Feng, and A. W. Poon, "Towards Adaptively Tuned Silicon Microring Resonators for Optical Networks - on - Chip Applications," IEEE J. Sel. Top. Quantum Electron. 20(4), 136–149 (2014).

[S30]. J. A. Cox, D. C. Trotter, and A. L. Starbuck, “Control of silicon-photonic micro-resonator wavelength via balanced homodyne locking,” Opt. Express 22(19), 12279–12289 (2014).
[S31]. X. Zheng, E. Chang, P. Amberg, I. Shubin, J. Lexau, F. Liu, H. Thacker, S. S. Djordjevic, S. Lin, Y. Luo, J. Yao, J.-H. Lee, K. Raj, R. Ho, J. E. Cunningham, and A. V. Krishnamoorthy, “A high-speed, tunable silicon photonic ring modulator integrated with ultra-efficient active wavelength control,” Opt. Express 22(10), 12628–12633 (2014).
